# Exosomes secreted from cancer-associated fibroblasts elicit anti-pyrimidine drug resistance through modulation of its transporter in malignant lymphoma

**DOI:** 10.1038/s41388-021-01829-y

**Published:** 2021-05-16

**Authors:** Shunsuke Kunou, Kazuyuki Shimada, Mika Takai, Akihiko Sakamoto, Tomohiro Aoki, Tomoya Hikita, Yusuke Kagaya, Eisuke Iwamoto, Masashi Sanada, Satoko Shimada, Fumihiko Hayakawa, Chitose Oneyama, Hitoshi Kiyoi

**Affiliations:** 1 Department of Hematology and Oncology, Nagoya University Graduate School of Medicine, Nagoya, Aichi Japan; 2 Fujii Memorial Research Institute, Otsuka Pharmaceutical Co., Ltd, Otsu, Shiga Japan; 3 Division of Cancer Cell Regulation, Aichi Cancer Center Research Institute, Nagoya, Aichi Japan; 4 Department of Target and Drug Discovery, Nagoya University Graduate School of Medicine, Nagoya, Aichi Japan; 5 Department of Advanced Diagnosis, Clinical Research Centre, National Hospital Organization Nagoya Medical Center, Nagoya, Aichi Japan; 6 Department of Pathology and Clinical Laboratories, Nagoya University Hospital, Nagoya, Aichi Japan

**Keywords:** Cancer microenvironment, B-cell lymphoma

## Abstract

The tumor microenvironment is deeply involved in the process of tumor growth and development. In this study, we focused on cancer-associated fibroblasts (CAFs) and their derived exosomes on the lymphoma microenvironment to uncover their clinical significance. CAFs were established from primary lymphoma samples, and exosomes secreted from CAFs were obtained by standard procedures. We then investigated the roles of CAFs and their derived exosomes in the survival and drug resistance of lymphoma cells. CAFs supported the survival of lymphoma cells through increased glycolysis, and the extent differed among CAFs. Exosomes were identified as a major component of the extracellular vesicles from CAFs, and they also supported the survival of lymphoma cells. The suppression of RAB27B, which is involved in the secretion of exosomes, using a specific siRNA resulted in reduced exosome secretion and decreased survival of lymphoma cells. Moreover, anti-pyrimidine drug resistance was induced in the presence of exosomes through the suppression of the pyrimidine transporter, equilibrative nucleoside transporter 2 (ENT2), and the suppression of ENT2 was significant in in vivo experiments and clinical samples. RNA sequencing analysis of miRNAs in exosomes identified miR-4717-5p as one of the most abundant miRNAs in the exosome, which suppressed the expression of ENT2 and induced anti-pyrimidine drug resistance in vitro. Our results suggest that exosomes including miR-4717-5p secreted from CAFs play a pivotal role in the lymphoma microenvironment, indicating that they are a promising therapeutic target.

## Introduction

Malignant lymphoma is the most common hematological malignancy but has heterogeneous phenotypes. In the last decade, progress has been made in the application of immunotherapies against common lymphocyte antigens such as CD20, CD19, CCR4, CD30, and PD1, which has significantly improved clinical outcomes [1–5]. However, about half of patients with malignant lymphoma show intractable disease; in particular, the prognoses of patients with insufficient responses to initial treatment are very poor [6]. Therefore, the appropriate choice of effective second-line treatment and the development of novel effective therapies for intractable patients are urgent issues [7].

The remarkable success of immunotherapies against malignant lymphoma indicates the importance of the tumor microenvironment (TME) [8]. Investigations of the TME have revealed that it contributes not only to tumorigenesis, but also to the maintenance of cancer stemness and treatment resistance [9, 10]. In particular, the extent of the infiltration of microenvironmental cells including lymphocytes and macrophages is associated with clinical outcomes [8, 11]. Cancer-associated fibroblasts (CAFs) are a key component of the TME, and many studies suggest that they play a prominent functional role in cancer progression and metastasis [12, 13]. We previously reported that CAFs can be isolated from primary lymph node samples of various lymphoma subtypes, and that they support lymphoma cell survival through the secretion of pyruvate leading to an increase of both anaerobic and aerobic metabolism of lymphoma cells [14, 15].

While metabolites play an important role in the TME, extracellular vesicles (EVs) have attracted attention for their roles in intercellular communication. In the TME, components of EVs are associated with various biological processes. Exosomes, a major component of EVs, are 30–150 nm diameter nanovesicles with endocytosis-induced phospholipid bilayer membranes. These nanovesicles can transport biologically active molecules, including miRNA, mRNA, proteins, and DNA from donor cells to recipient cells, and are involved in the promotion of tumor cell proliferation, metastasis, and immune evasion [9]. For example, previous reports have revealed that exosomes derived from tumor cells promote angiogenesis by acting on vascular endothelial cells [16, 17] and act on immune cells to suppress their response to tumor cells [16, 18]. However, among studies on exosomes derived from the TME, those focused on fibroblasts-derived exosomes are relatively limited. In breast cancer, fibroblast-derived exosomes promote metastasis by activating Wnt-PCP signaling in tumor cells [19]. In pancreatic ductal adenocarcinomas, drug resistance of tumor cells is induced by CAFs via exosomes [20]. These findings suggest that exosomes from CAFs in malignant lymphoma might also be important; however, their roles remain unknown. In this study, we examined the role of exosomes derived from CAFs in malignant lymphoma and their significance as a potential therapeutic target.

## Materials and methods

### Patients and their samples

All patient samples and information were collected from patients receiving organ biopsies at Nagoya University Hospital who were diagnosed with malignant lymphoma (Table S1). The study protocol for the experimental use of patient samples and information was approved by the Institutional Review Board of Nagoya University Hospital (approval number: 1357 and 2014-0081) and complied with all provisions of the Declaration of Helsinki and the Ethical Guidelines issued by the Ministry of Health, Labour, and Welfare in Japan. All live cell samples from patients were used experimentally after obtaining written informed consent.

### Tumor cells from patient-derived xenograft models and CAFs

Tumor cells from patient-derived xenograft (PDX) models and CAFs were obtained as described previously [14, 15, 21, 22]. Detailed procedures are described in the supplemental methods.

### Drugs

Gemcitabine hydrochloride, cytarabine, and bendamustine hydrochloride hydrate were purchased from Tokyo Chemical Industry Co., Ltd. (Tokyo, Japan). Doxorubicin hydrochloride was purchased from FUJIFILM Wako Pure Chemical Corporation (Osaka, Japan). Cytarabine-13C3 was purchased from Santa Cruz (Dallas, TX, USA).

### Isolation of tumor cells

To purify B-cell lymphoma cells, cells from primary lymph node samples or PDX models were magnetically isolated by using CD19 beads (Miltenyi Biotec, Bergisch Gladbach, Germany).

### Cell lines

SU-DHL4 and SU-DHL6 were used as representative follicular lymphoma (FL) cell lines. OCI-Ly3 and OCI-Ly10 were used as representative diffuse large B-cell lymphoma (DLBCL) cell lines. SU-DHL4, OCI-Ly3, and OCI-Ly10 were kindly gifted from Dr. Kunihiko Takeyama (Dana Farber Cancer Institute, Boston, MA, USA) previously. SU-DHL6 was obtained from the ATCC (Manassas, VA, USA). Culture conditions are described in the supplemental methods.

### Exosome purification

One million CAFs were cultured on a 10-cm dish for 24 h with IMDM supplemented with 10% fetal bovine serum (FBS). Subsequently, cells were washed with phosphate-buffered saline (PBS) and then cultured with IMDM supplemented with 10% exosome-free FBS from which exosomes had been removed by ultracentrifugation at 32,000 rpm for 16 h (ultracentrifuge: Optima LE-80 or Optima L-100, Beckman Coulter, CA, USA; ultracentrifuge rotor: SW32Ti-12U, Beckman Coulter). After incubation for 48 h, conditioned medium (CM) was collected and centrifuged at 300 × *g* for 10 min at 4 °C. Then the supernatant was centrifuged at 2000 × *g* for 10 min at 4 °C. To thoroughly remove cellular debris, the supernatant was passed through a 0.22-μm filter. The CM was ultracentrifuged at 35,000 rpm using a SW41Ti-14E2457 rotor for 70 min at 4 °C or at 32,000 rpm using a SW32Ti-12U rotor for 84 min at 4 °C. The pellets containing exosomes were washed with 0.22-μm membrane-filtered PBS. PBS containing exosomes was ultracentrifuged at 35,000 rpm using the SW41Ti-14E2457 rotor for 70 min at 4 °C or at 32,000 rpm using a SW32Ti-12U rotor for 84 min at 4 °C. Finally, the pellets containing exosomes were resuspended in 0.22-μm membrane-filtered PBS.

### Cell viability and cytotoxicity assessment

To assess the cell viability and cytotoxicity of lymphoma cells co-cultured with CAFs, the death of lymphoma cells in the presence of exosomes derived from CAFs, and tumor cell death, we used an image analyzer, WST-assays, and propidium iodide (PI) or 7-aminoactinomycin D (7-AAD) and Annexin V-fluorescein isothiocyanate (FITC) assays, respectively. Detailed procedures are described in the supplemental methods.

### Nanoparticle tracking analysis

The concentration of exosomes was measured by nanoparticle tracking analysis (NTA) using a NanoSight LM10 instrument (Malvern Panalytical, Malvern, UK) with NTA3.1 software. Thirty-second measurements were recorded for five times for each sample. The camera level was set at 14 and the detection threshold at 10.

### Transmission electron microscopy

To observe exosomes by electron microscopy, they were adsorbed to a carbon-coated copper grid (NISSHIN EM, Tokyo, Japan) and then stained with uranyl acetate. The samples were then observed with a transmission electron microscope (JEM-1400Plus, JEOL Ltd., Tokyo, Japan).

### Cellular uptake of exosomes

To observe the cellular uptake of exosomes, they were labeled with PKH26 (Sigma-Aldrich, St. Louis, MO, USA). A mixture of exosomes and PKH26 solution was incubated for 5 min at room temperature and then put in a Vivacon 500/100k filter unit (Sartorius, Gottingen, Germany) followed by centrifugation at 14,000 × *g* for 2 min at room temperature. The pellet containing PKH26-labeled exosomes was washed with PBS four times. PKH26-labeled exosomes were added into medium in which Hoechst 33342 (Invitrogen, Thermo Fisher Scientific, Carlsbad, CA, USA)-labeled lymphoma cells were cultured, and then incubated on 35-mm glass base dishes (Iwaki, Shizuoka, Japan) for 12 h at 37 °C. Cellular uptake of exosomes was observed with a confocal fluorescence microscope (TiE-A1R, Nikon, Tokyo, Japan).

### Measurement of adenosine triphosphate production

Measurement of adenosine triphosphate (ATP) production was performed as described previously [14]. In brief, the ATP concentration was assessed using a Colorimetric ATP Assay Kit (Abcam, Cambridge, UK) according to the manufacturer’s protocol.

### Metabolome analysis

To analyze the comprehensive metabolites of tumor cells in the presence or absence of CAF or CAF-derived exosomes, metabolic analyses were carried out. Metabolite analyses using capillary electrophoresis-time-of-flight mass spectrometry (CE-TOFMS) and capillary electrophoresis tandem mass spectrometry (CE-QqQMS) were carried out by Human Metabolome Technologies (Tsuruoka, Japan).

### Quantitative real-time reverse transcriptase (RT)-PCR

To quantitate the gene expression of *SLC29A2*, quantitative RT-PCR was carried out. Detailed procedures are described in the supplemental methods.

### Immunoblotting

Immunoblotting was performed as described previously [23, 24]. In brief, cells were treated with the indicated drug and lysed. Samples were separated by sodium dodecyl sulfate polyacrylamide gel electrophoresis and transferred to polyvinylidene difluoride membranes that were then blocked with 5% skimmed milk in TBS-Tween buffer (50 mM Tris-HCL [pH 7.4], 150 mM NaCl, and 0.05% Tween 20). Immunoblotting was carried out using primary antibodies (Table S2) appropriately diluted in TBS-Tween buffer containing 5% BSA and 0.05% sodium azide. Signals were detected with the appropriate horseradish peroxidase-conjugated secondary antibodies appropriately diluted in TBS-Tween buffer. Images were visualized with a LAS-4000 mini-image analyzer (FUJIFILM, Tokyo, Japan) and analyzed with MultiGauge software (FUJIFILM).

### Knockdown of an exosome secretion-associated protein with small interfering RNA

To evaluate the significance of exosome secretion, knockdown of RAB27B was performed [25]. CAFs were transfected with small interfering RNAs (siRNAs) for *RAB27B* using Lipofectamine RNAiMAX reagent (Invitrogen) according to the manufacturer’s protocol. The siRNA for *RAB27B* was purchased from Theoria Science, Tokyo, Japan. One hundred fifty thousand CAFs were seeded on 6-well plates and then incubated for 24 h. A mixture of Lipofectamine RNAiMAX reagent and siRNA for *RAB27B* was added to each well for transfection and then incubated at 37 °C for 48 h. Transfected CAFs were lysed to generate protein for immunoblotting. Exosomes were isolated from the CM of transfected CAFs by ultracentrifugation, and particle counts of exosomes were made with the NTA assay.

### Quantitation of gemcitabine-triphosphate and ara-cytidine-5′-triphosphate in lymphoma cells

To evaluate the intracellular concentrations of gemcitabine and cytarabine active metabolites, gemcitabine-triphosphate (GEM-TP) and ara-cytidine-5′-triphosphate (Ara-CTP) were measured by liquid chromatography/tandem mass spectrometry (LC-MS/MS). After the culturing of lymphoma cells with CAFs or in the presence of exosomes for 48 h, 1 × 10^6^ lymphoma cells were incubated with 200 nM gemcitabine or 200 nM cytarabine-13C3 for 4 h. Cells were then harvested to obtain cell extracts. In brief, cells were washed with ice-cold 5% mannitol, and pellets were obtained by centrifugation. Ice-cold 500 μl methanol and 250 μl water were added to cell pellets, which were then vortexed for 3 s. Five hundred and fifty microliters of the mixture was then centrifuged at 10,000 × *g* for 10 min at 4 °C. Four hundred microliters of supernatant were collected and dried with a centrifugal evaporator (Labconco7810010, Asahi Life Science, Saitama, Japan) for 90 min at 40 °C. Two hundred microliters of 10 mM ammonium bicarbonate pH 9.4 were added to the dried cell extracts, the internal standard solution was added, and the mixture solution was sonicated and vortexed followed by centrifugation at 10,000 rpm for 10 min at 4 °C. The supernatant was analyzed with a Triple Quad 5500 (SCIEX, Tokyo, Japan).

### RNA sequencing

To analyze miRNAs in the CAF-derived exosomes, RNA sequencing was carried out. In brief, miRNA was extracted from exosomes with an miRNeasy mini kit according to the manufacturer’s protocol (Qiagen, Venlo, Netherlands). The quality and quantity of miRNAs were evaluated using an Agilent RNA6000 pico kit with an Agilent 2100 bioanalyzer (Agilent Technologies, Santa Clara, CA, USA). A miRNA library for RNA sequencing was prepared using a TruSeq Small RNA Library Preparation Kit (Illumina, San Diego, CA, USA). The library was sequenced with an Illumina MiSeq system in 51-base pair single-end reads (Illumina). MiRNA read counts were obtained by mapping to 2588 mature miRNAs using Illumina miRNAs analysis application ver. 0.9.30 (Illumina).

### In vivo studies

To evaluate gemcitabine resistance induced by CAFs in vivo, 1 × 10^7^ tumor cells from patients with or without 4 × 10^6^ CAFs were subcutaneously inoculated into the flanks of NOD/Shi-*scid* IL2Rγ^null^ (NOG) male mice (7 to 8 weeks old) (purchased from In-Vivo Science Inc. Tokyo, Japan). Treatment was initiated when the inoculated tumors reached a size of at least 300 mm^3^, defined as day 0. Mice were intraperitoneally treated with gemcitabine on days 1, 3, and 5. Tumor volume was measured every other day and was calculated using the following formula: tumor volume (mm^3^) = 4/3 × π × (*d*/2)^2^ × (*D*/2), where *D* (mm) and *d* (mm) are the longest and shortest diameters of the tumor, respectively. All mice treated with gemcitabine were sacrificed on day 12. Untreated control mice were sacrificed for ethical considerations when the tumor size exceeded 2500 mm^3^. The sample sizes of mice in each experimental group were not based on statistical methods. Mice were randomly assigned to experimental groups and evaluations of animal studies were not blinded. All the animal experimental procedures complied with the Regulations regarding Animal Experiments in Nagoya University.

### Pathological analyses, immunohistochemical staining, and TUNEL staining

The formalin-fixed, paraffin-embedded tissues of patient and mouse samples were evaluated using routine hematoxylin-eosin (HE) and immunohistochemical (IHC) staining [24]. For IHC of equilibrative nucleoside transporter 2 (ENT2), which is encoded by the *SLC29A2* (solute carrier family 29 member 2), and terminal deoxynucleotidyl transferase dUTP nick end labeling (TUNEL) staining, after deparaffinization and rehydration of the sections, antigen retrieval was performed with Target Retrieval Solution, Citrate pH 6 (Dako, Glostrup, Denmark) for 10 min at 98 °C using a microwave oven. The sections were subsequently incubated with primary antibody for ENT2 at room temperature for 75 min followed by the addition of biotin-conjugated secondary antibody for 30 min at room temperature. Staining was activated by the addition of the avidin-biotin complex. Horseradish peroxidase activity was detected with 3,3-diaminobenzidine tetrahydrochloride. For TUNEL staining, the sections were incubated with TUNEL reaction mixture (Roche, Basel, Switzerland) for 60 min at 37 °C. The specimens were observed, and photographs were taken with a BZ9000 (Keyence, Osaka, Japan).

### Analysis of patients receiving anti-pyrimidine drugs

To examine the clinical significance of the anti-pyrimidine drug transporter protein ENT2, pathological specimens of relapsed or refractory patients receiving the gemcitabine-containing treatment GCDR (gemcitabine, carboplatin, dexamethasone, and rituximab) [26] and lymph node biopsies from 1 January 2012 to 31 August 2018 were analyzed. A clinical response to GCDR was defined as follows: a responder was a patient who displayed a treatment response more than stable disease for at least four courses of GCDR, and non-responder was defined as a patient who displayed disease progression within three courses of GCDR according to the revised response criteria [27].

### Statistical analysis

All quantitative results are presented as the mean ± standard error of the mean taken from more than three independent experiments. The statistical significance of in vitro experiments was evaluated by an unpaired *t* test to compare two groups of independent samples, by one-way ANOVA or two-way ANOVA to compare multiple groups, and *P* < 0.05 was considered significant. All statistical analyses were performed using GraphPad Prism Versions 7 and 8 (GraphPad Software Inc., La Jolla, CA, USA) or SAS 9.4 (SAS Institute Inc., Cary, NC, USA).

## Results

### CAFs support the survival of primary lymphoma cells by enhancing glycolysis

**Fig. 1:**
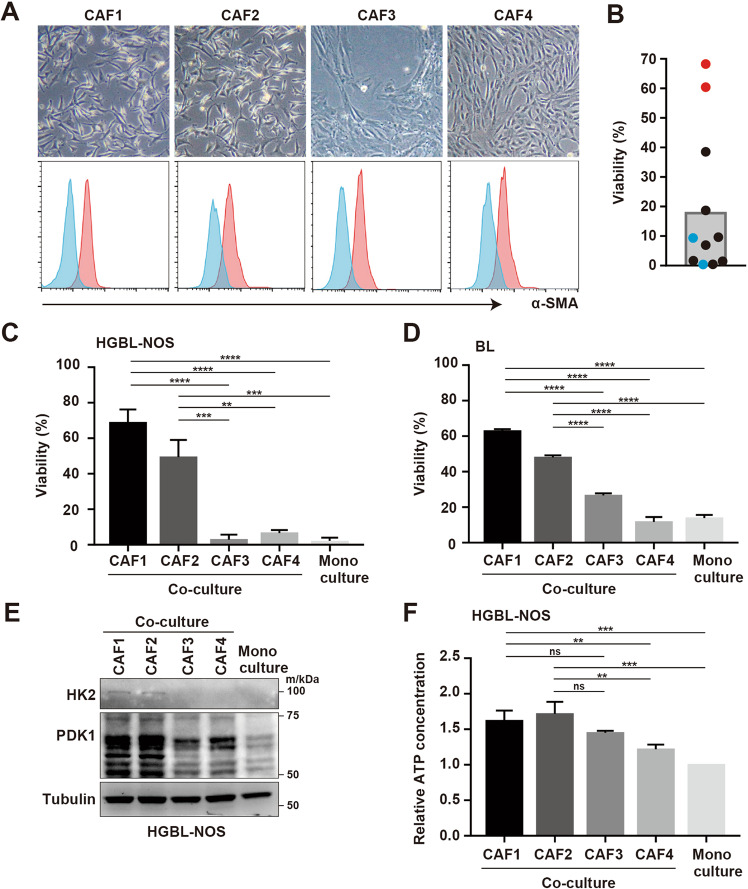
Diversity of survival of lymphoma cells with CAFs. **A** Phase-contrast images and the expression of α-SMA via FCM of CAF1, CAF2, CAF3, and CAF4. **B** Viability of HGBL-NOS cells co-cultured with each CAF. CAFs that displayed strong improvement of HGBL-NOS survival (CAF1 and CAF2) (red circles) and weak improvement (CAF3 and CAF4) (blue circles) and high proliferation potential were selected. Each point represents the viability of HGBL-NOS cells co-cultured with each CAF, and the bar represents the mean value. **C** Viability of tumor cells from patients diagnosed with HGBL-NOS in monoculture and co-culture with CAF1 to CAF4. **D** Viability of tumor cells from patients diagnosed with BL in monoculture and co-culture with CAF1 to CAF4. **E** Immunoblotting for HK2, PDK1, and tubulin as a loading control in HGBL-NOS cells in monoculture and co-culture with CAF1 to CAF4. **F** Relative ATP concentrations of HGBL-NOS cells in monoculture and co-culture with CAF1 to CAF4. Asterisks indicate *p* values as follows: *0.05 > *p* ≥ 0.01, **0.01 > *p* ≥ 0.001, ***0.001 > *p* ≥ 0.0001, ****<0.0001. Each bar is the mean value taken from 3 or more independent experiments with error bars indicating standard error. Ns, not significant.

We successfully isolated fibroblasts from 12 patient biopsy samples of various types of malignant lymphomas such as FL and DLBCL (Table S3). Isolated fibroblasts were positive for α-SMA according to FCM, indicating that this characteristic was coincident with CAFs (Fig. 1A). We previously reported that CAFs can support primary lymphoma cells [14, 15], so we examined whether CAFs could support lymphoma cells from the PDX model established using high-grade B-cell lymphoma–not otherwise specified (HGBL-NOS). Intriguingly, the extent of support for lymphoma cell survival differed among CAFs, so we picked four representative CAFs established from FL, DLBCL, and T lymphoblastic lymphoma (T-LBL) patients, which strongly or weakly supported the survival of lymphoma cells (Fig. 1B). In co-cultures with CAF1 established from FL, and CAF2 established from T-LBL, lymphoma cells from PDX models (HGBL-NOS and Burkitt lymphoma [BL]) displayed higher viability compared with monoculture. In addition, lymphoma cells co-cultured with CAF1 and CAF2 survived significantly better compared with those with CAF3 and CAF4, both established from DLBCL, irrespective of lymphoma subtype (Fig. 1C, D, and Fig. S1A–E). These two lymphoma cell lines from PDX models were mainly used in subsequent analyses. Subsequently, we compared glycolysis activity of lymphoma cells co-cultured with CAFs. In lymphoma cells co-cultured with CAF1 and CAF2, the expressions of HK2 and PDK1, key enzymes of glycolysis, were higher than in those with CAF3 and CAF4 (Fig. 1E). Moreover, ATP production was more increased under co-cultures with CAF1 and CAF2 compared with monocultures, suggesting that lymphoma cells co-cultured with CAF1 and CAF2 had increased glycolysis (Fig. 1F).

### Exosomes as EVs secreted from CAFs

**Fig. 2:**
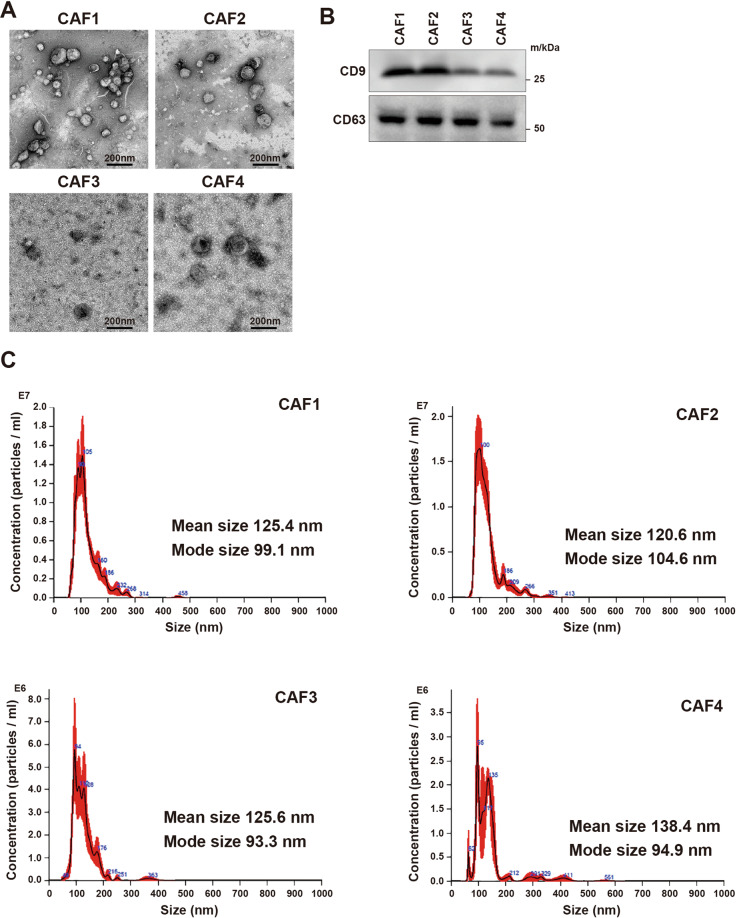
Extracellular vesicles from CAFs. **A** Electron microscopic images of extracellular vesicles from each CAF. Scale bar, 200 nm. **B** Immunoblotting for CD9 and CD63 for each CAF. **C** Nanoparticle tracking analysis for CAF1, CAF2, CAF3, and CAF4.

**Fig. 3:**
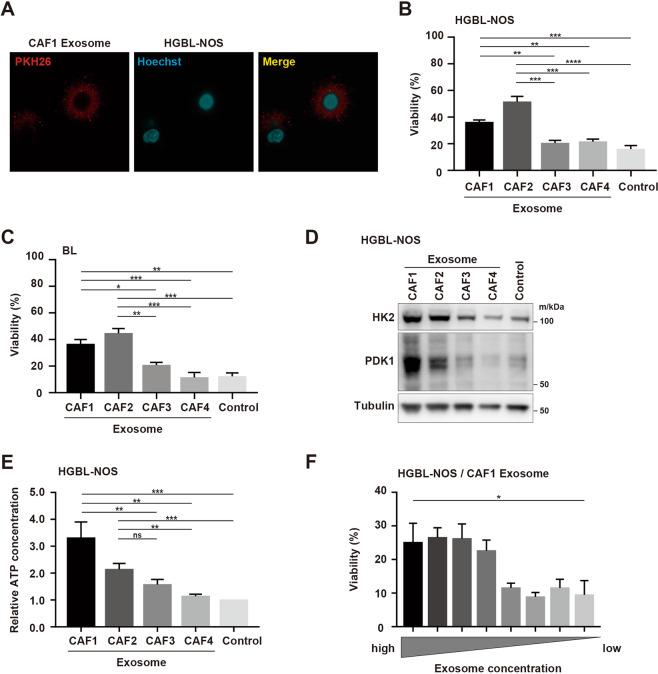
Survival of lymphoma cells in the presence of exosomes from CAFs. **A** Fluorescent microscopic image of exosomes stained with PKH26 (red), tumor cells stained with Hoechst 33342 (blue), and a merged image. **B** Viability of HGBL-NOS cells in monoculture and in the presence of exosomes from each CAF. Ten thousand tumor cells were seeded in 96-well plates with 0.8 × 10^9^ exosomes, and then viability was evaluated after 48-h incubation. **C** Viability of BL cells in monoculture and in the presence of exosomes from each CAF. The conditions were the same as in (**B**). **D** Immunoblotting for HK2, PDK1, and tubulin as a loading control in HGBL-NOS cells in monoculture and in the presence of exosomes. Three hundred thousand tumor cells were seeded in 6-well plates with 2.4 × 10^10^ exosomes, and then proteins were obtained after 24-h incubation. **E** Relative ATP concentrations of HGBL-NOS cells in the presence of exosomes from each CAF. Three hundred thousand tumor cells were seeded in 6-well plates with 2.4 × 10^10^ exosomes, and then assays were performed after 24-h incubation. **F** Viability of HGBL-NOS cells in the presence of various concentrations of exosomes from CAF1. The highest exosome concentration was 2.5 × 10^10^ particles/ml and was serially diluted 2-fold to give a concentration gradient. Asterisks indicate *p* values as follows: *0.05 > *p* ≥ 0.01, **0.01 > *p* ≥ 0.001. Each bar is the mean value taken from 3 or more independent experiments with error bars indicating standard error. Ns, not significant.

Based on the assumption that secretions from CAFs other than metabolites could support lymphoma cells in the TME of malignant lymphoma, we focused on EVs from CAFs. In isolated EVs, vesicles less than 200 nm were observed by electronic microscopy (Fig. 2A). Those vesicles were positive for CD9 and CD63, which are surface antigens used as exosome markers (Fig. 2B). In addition, the mean sizes of vesicles from all CAFs were 120 to 140 nm, which is characteristic of exosomes (Fig. 2C). We then examined the function of exosomes from CAFs. First, we confirmed that exosomes stained with PKH26 were endocytosed into lymphoma cells (Figs. 3A and S2A). In the presence of exosomes secreted from CAF1 and CAF2, which showed strong support for lymphoma cells in co-culture, the survival of lymphoma cells was better supported compared with exosomes from CAF3 and CAF4 (Fig. 3B, C). The expressions of HK2 and PDK1 in the presence of exosomes from CAF1 and CAF2 were higher than those from CAF3 and CAF4, indicating that glycolysis in lymphoma cells was increased in the presence of exosomes from CAF1 and CAF2 (Figs. 3D and S2B). ATP production was also more increased in the presence of exosomes from CAF1 and CAF2 (Fig. 3E). Moreover, the support from exosomes was better with a higher concentration of exosomes (Figs. 3F and S2C). Metabolomic analysis indicated that the glucose 6-phosphate/ribose 5-phosphate ratio (G6P/R6P ratio), which is parameter of glycolytic activity, was higher in tumor cells in the presence of exosomes from CAF1 or in those co-cultured with CAF1, demonstrating increased glycolysis (Fig. S3 and Table S4). Collectively, these data indicated that exosomes secreted from CAFs were at least in part involved in the improved survival of lymphoma cells by CAFs via increased glycolysis.

### Reversal of improved survival by attenuating exosome secretion

**Fig. 4:**
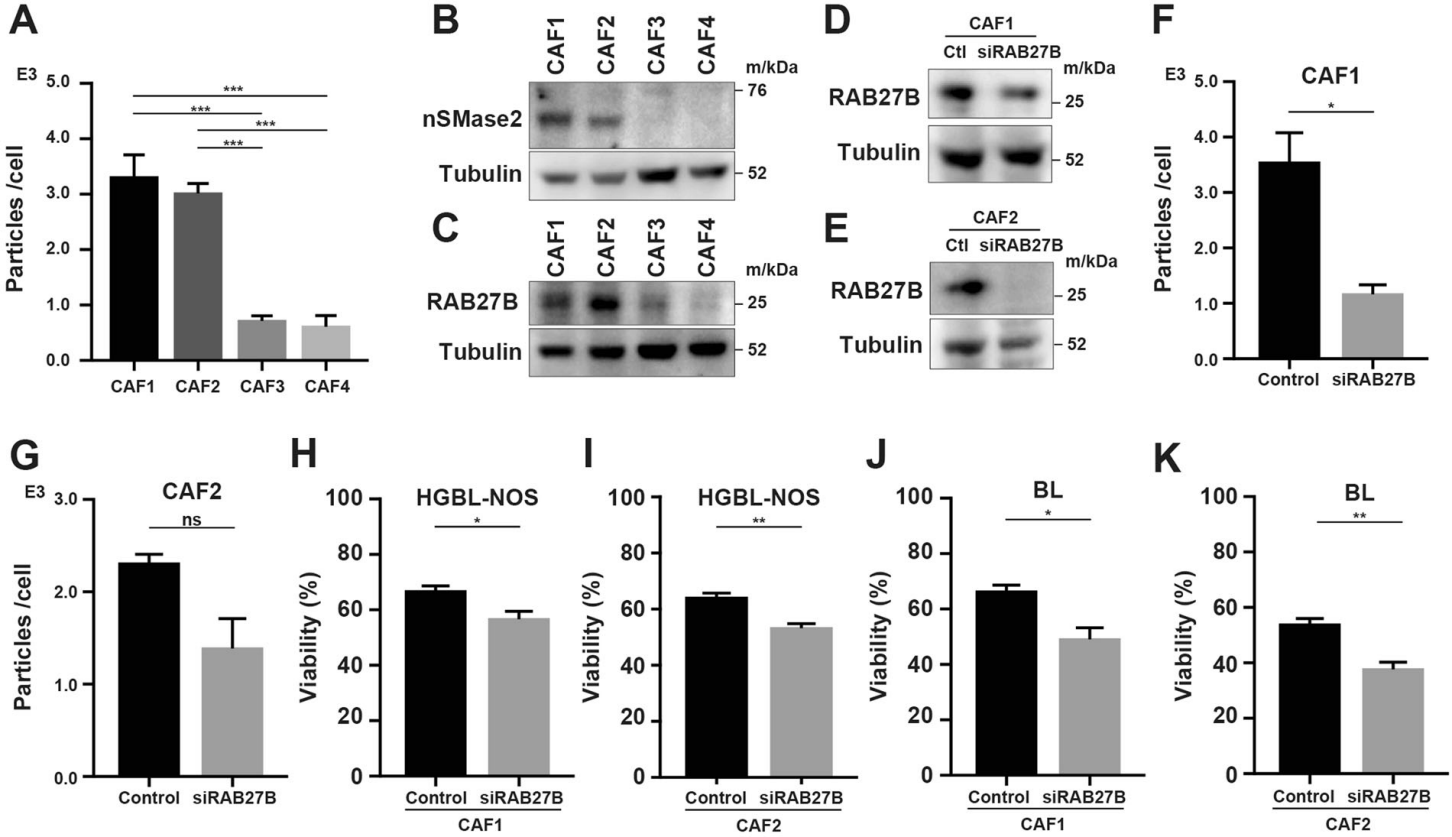
Amounts of exosome secretion from each CAF. **A** Number of particles secreted from each CAF as measured by NTA. **B** Immunoblotting for nSMase2 and tubulin in each CAF. **C** Immunoblotting for RAB27B and tubulin in each CAF. **D**, **E** Immunoblotting for RAB27B and tubulin in CAF1 and CAF2 transfected with siRNA as indicated. **F**, **G** Number of particles secreted from CAF1 and CAF2 transfected with siRNA as indicated. **H**–**K** Viability of HGBL-NOS and BL cells co-cultured with CAFs transfected with siRNA as indicated. Asterisks indicate *p* values as follows: *0.05 > *p* ≥ 0.01, **0.01 > *p* ≥ 0.001. Each bar is the mean taken from three or more independent experiments with error bars indicating standard error. Ns, not significant.

Next, exosomes from each CAF were quantified by NTA since their effects differed. The amount of exosomes from each CAF corresponded to the extent of the improved survival (Fig. 4A). We further analyzed the relationship between the exosome amount with the expressions of neutral sphingomyelinase 2 (nSMase2), which is an enzyme that synthesizes ceramide, and RAB27B, which is an exosome secretion-associated protein of the small Rab GTPase family [25, 28]. The expressions of these proteins by CAF1 and CAF2 were higher than those of CAF3 and CAF4 (Fig. 4B, C). Next, decreased secretion of exosomes was observed by reducing RAB27B expression using a specific siRNA (Fig. 4D–G). In addition, the survival of lymphoma cells was significantly decreased in co-culture with *RAB27B* knocked-down CAFs (Fig. 4H–K). Together, these data indicated that the improved survival by CAFs was decreased by reducing the secretion of their derived exosomes.

### CAFs induce drug resistance in lymphoma cells via exosomes

**Fig. 5:**
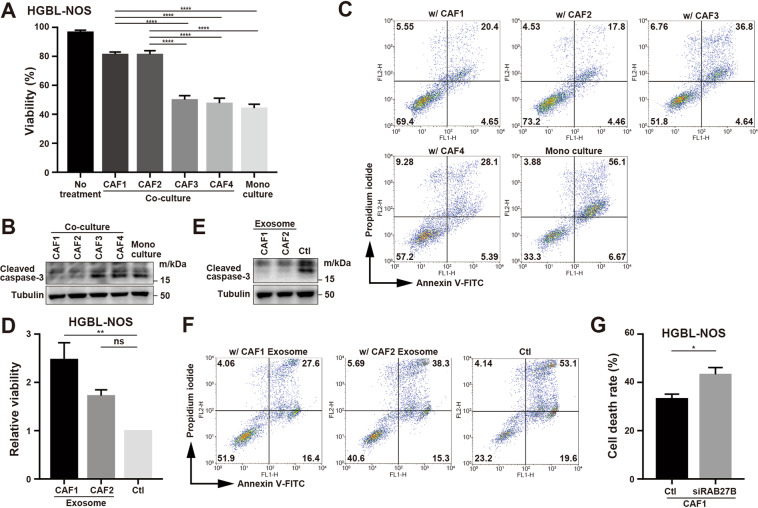
Change of susceptibility to anti-pyrimidine drugs induced by CAFs and their derived exosomes. **A** Viability of HGBL-NOS cells in monoculture and co-culture with CAF1 to CAF4 in the presence of 10 nM gemcitabine. **B** Immunoblotting for cleaved caspase-3 and tubulin as a loading control in HGBL-NOS cells in the above conditions. **C** Susceptibility of HGBL-NOS cells to gemcitabine. Assessment of cell death of HGBL-NOS cells in monoculture and co-culture with each CAF is shown. **D** Relative viability of HGBL-NOS cells to 10 nM gemcitabine in the presence of exosomes from CAF1 and CAF2. Ten thousand tumor cells were seeded in 96-well plates with 5.0 × 10^9^ exosomes and gemcitabine, and then viability was evaluated after 48-h incubation. **E** Immunoblotting for cleaved caspase-3 and tubulin as a loading control in HGBL-NOS cells in the presence of 10 nM gemcitabine. Three hundred thousand tumor cells were seeded in 6-well plates with 1.5 × 10^11^ exosomes, and proteins were obtained after 24-h incubation. **F** Assessment of cell death of HGBL-NOS cells treated with 10 nM gemcitabine in the presence of exosomes from CAF1 and CAF2. The conditions were the same as in (E). Cell death was evaluated after 48-h incubation. **G** Cell death of HGBL-NOS cells co-cultured with CAF1 transfected with siRNA for RAB27B. Asterisks indicate *p* values as follows: *0.05 > *p* ≥ 0.01, **0.01 > *p* ≥ 0.001, ***0.001 > *p* ≥ 0.0001. Each bar is the mean taken from three or more independent experiments with error bars indicating standard error.

**Fig. 6:**
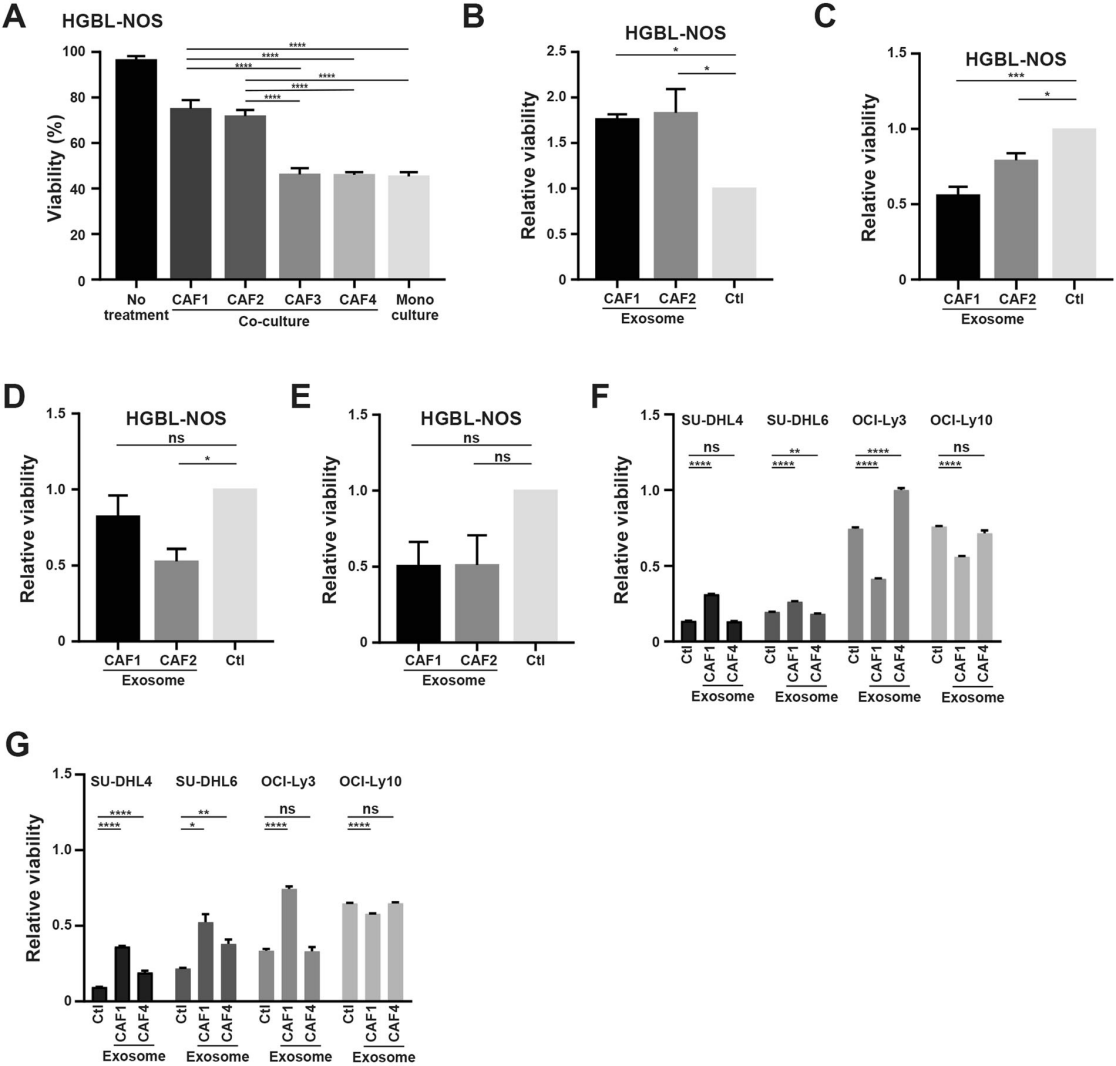
Susceptibility to anti-cancer drugs induced by CAFs and their derived exosomes. **A** Viability of HGBL-NOS cells in monoculture and co-culture with CAF1 to CAF4 in the presence of 100 nM cytarabine. **B** Relative viability of HGBL-NOS cells. Assessment of cell death of HGBL-NOS cells to cytarabine in the presence of exosomes from CAF1 and CAF2 is shown. **C**–**E** Relative viabilities of HGBL-NOS cells to 25 μM bendamustine (**C**), 100 nM doxorubicin (**D**), and 5 nM vincristine (**E**) in the presence of exosomes from CAF1 and CAF2. Ten thousand tumor cells were seeded in 96-well plates with 5.0 × 10^9^ exosomes, and then viability was evaluated after 48-h incubation. **F**, **G** Relative viability of representative FL cell lines and DLBCL cell lines exposed to gemcitabine (20 nM in SU-DHL4, SU-DHL6, and OCI-Ly10, and 1 nM in OCI-Ly3) (**F**) and cytarabine (1250 nM in SU-DHL4 and SU-DHL6, 25 nM in OCI-Ly3, and 1000 nM in OCI-Ly10) (**G**) in the presence of CAF1- and CAF4-derived exosomes. One hundred and twenty-five thousand tumor cells were seeded in 96-well plates with 8.0 × 10^9^/mL particles of exosomes, and then viability was evaluated after 48-h incubation. Asterisks indicate *p* values as follows: *0.05 > *p* ≥ 0.01, **0.01 > *p* ≥ 0.001, ***0.001 > *p* ≥ 0.0001, ****<0.0001. Each bar is the mean taken from three or more independent experiments with error bars indicating standard error. Ns, not significant.

To examine the clinical significance of improved survival from CAFs or their derived exosomes, we studied the effectiveness of anti-cancer drugs in the presence of CAFs or exosomes. Under treatment by gemcitabine, the survival of lymphoma cells co-cultured with CAF1 and CAF2 was better supported than that with CAF3 or CAF4 (Figs. 5A–C, S4A and S4B). In the presence of exosomes derived from CAF1 and CAF2, the survival of lymphoma cells was also improved (Figs. 5D and S4C), and cell death was significantly suppressed (Figs. 5E, F, and S4D). Under suppressed secretion of exosomes by reducing RAB27B expression using siRNA, the susceptibility of lymphoma cells to gemcitabine was partially restored (Figs. 5G, S4E–G). Collectively, these data indicated that exosomes derived from CAFs were involved in the resistance to gemcitabine by lymphoma cells. Next, we examined whether anti-cancer drugs other than gemcitabine could be involved in exosome-induced changes in chemosensitivity. In the presence of cytarabine or bendamustine, susceptibility of lymphoma cells to these drugs was decreased under co-culture with CAFs (Figs. 6A, S5A, B). In the presence of exosomes from CAFs, decreased susceptibility to cytarabine was observed (Figs. 6B and S5C), but susceptibility to bendamustine, doxorubicin, or vincristine was not affected (Fig. 6C–E). Taken together, we concluded that exosomes derived from CAFs could induce resistance to gemcitabine and cytarabine.

To analyze whether the origin of lymphoma cells and CAFs affects survival and the induction of drug resistance of tumor cells, we studied the association between representative FL cell lines (SU-DHL4 and SU-DHL6) and DLBCL cell lines (OCI-Ly3 and OCI-Ly10) and CAFs derived from corresponding disease types (CAF1 and CAF4), respectively. In co-cultures of cell lines with CAFs, the survival of the cell line and CAFs were not well maintained in OCI-Ly3 (Fig. S5D). We instead evaluated the acquisition of drug resistance of SU-DHL4, SU-DHL6, OCI-Ly3, and OCI-Ly10 in the presence of CAF1- or CAF4-derived exosomes. Gemcitabine and cytarabine resistances in representative FL cell lines were induced in the presence of exosomes from FL-derived CAFs. However, the acquisition of drug resistance in DLBCL cell lines was induced in the presence of exosomes from DLBCL-derived CAFs, particularly in OCI-Ly3 exposed to gemcitabine (Fig. 6F, G). Together these data indicated that drug resistance in cell lines tended to be induced in the presence of CAF-derived exosomes with similar cells of origin.

### Inhibition of drug uptake associated with gemcitabine and cytarabine resistance by CAF-derived exosomes

**Fig. 7:**
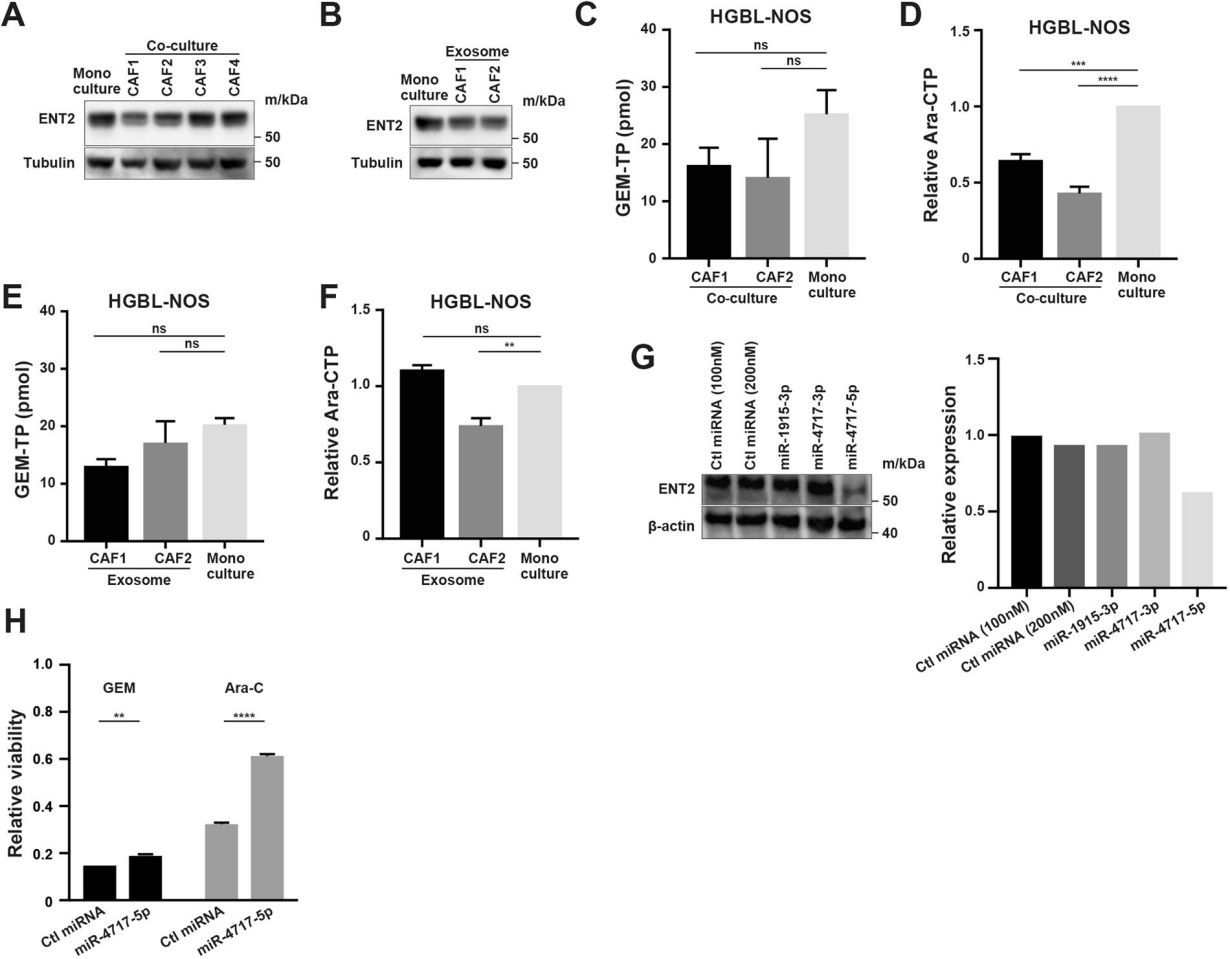
Expression of an anti-pyrimidine drug transporter and intracellular concentrations of drugs. **A** Immunoblotting for ENT2 and tubulin as a loading control in HGBL-NOS cells in monoculture and co-culture with each CAF. **B** Immunoblotting for ENT2 and tubulin as a loading control in HGBL-NOS cells in the presence of exosomes from CAF1 and CAF2. Three hundred thousand tumor cells were seeded in 6-well plates with 15 × 10^10^ exosomes, and proteins were obtained after 24-h incubation. **C**, **D** Intracellular concentrations of GEM-TP (**C**) and Ara-CTP (**D**). HGBL-NOS cells in the presence of 200 nM gemcitabine (**C**) and 200 nM cytarabine (**D**) in monoculture and co-culture with CAF1 and CAF2 were evaluated. **E**, **F** Intracellular concentrations of gemcitabine (**E**) and cytarabine (**F**) in HGBL-NOS cells in the presence of exosomes from CAF1 and CAF2. **G** Immunoblotting for ENT2 and β-actin as a loading control in HGBL-NOS cells transfected with each miRNA (left). Relative expression of ENT2 in immunoblotting (right). **H** Relative viability of HGBL-NOS cells transfected with each miRNA in the presence of 20 nM gemcitabine and 5 μM cytarabine. Asterisks indicate *p* values as follows: *0.05 > *p* ≥ 0.01, **0.01 > *p* ≥ 0.001, ****<0.0001. Each bar is the mean taken from 3 or more independent experiments with error bars indicating standard error. Ns, not significant.

Gemcitabine and cytarabine are taken into cells through their transporter, ENT2. To uncover the mechanism of action of exosome-induced chemoresistance, the expression of ENT2 was examined. In lymphoma cells co-cultured with CAFs, ENT2 expression was suppressed (Fig. 7A and S6A). In the presence of exosomes derived from CAF1 and CAF2, ENT2 expression in lymphoma cells was also suppressed (Figs. 7B and S6B). Intracellular concentrations of gemcitabine and cytarabine in lymphoma cells were then examined. In lymphoma cells co-cultured with CAFs, the intracellular concentration of GEM-TP tended to be lower than that in monoculture, and the concentration of Ara-CTP in lymphoma cells co-cultured with CAFs was significantly lower than that in monoculture (Figs. 7C, D, S6C, D). In the presence of exosomes derived from CAF1 and CAF2, intracellular concentrations of GEM-TP and Ara-CTP in lymphoma cells were lower than those in the absence of exosomes (Figs. 7E, F, S6E, F). Collectively, these data indicated that CAF-derived exosome-induced chemoresistance to gemcitabine and cytarabine was at least in part due to the decreased expression of their transporter protein, ENT2.

### MiR-4717-5p contained in exosomes is responsible for the suppression of ENT2

We subsequently analyzed the miRNAs contained in exosomes with miRNA-seq to uncover the mechanism of the suppression of ENT2. We identified three miRNAs with read counts more than 100 in at least either CAF1 or CAF2: miR-1915-3p and miR-4717-3p, which were predicted to target *SLC29A2* in the database (http://www.targetscan.org/vert_72/), and miR-4717-5p, which was the most abundant miRNA in CAF1 and the third most in CAF2 (Table S5). As expected, in the presence of miR-4717-5p, the mRNA expression of *SLC29A2* was not changed (Fig. S6G), but the expression of ENT2 was decreased (Fig. 7G). In addition, tumor cells with introduced miR-4717-5p acquired resistance to gemcitabine and cytarabine (Fig. 7H). These data suggested that miR-4717-5p in CAFs, which is translocated to tumor cells via exosomes, at least in part induced the suppression of ENT2, leading to the resistance to anti-pyrimidine drugs.

### In vivo drug resistance induced by CAFs

**Fig. 8:**
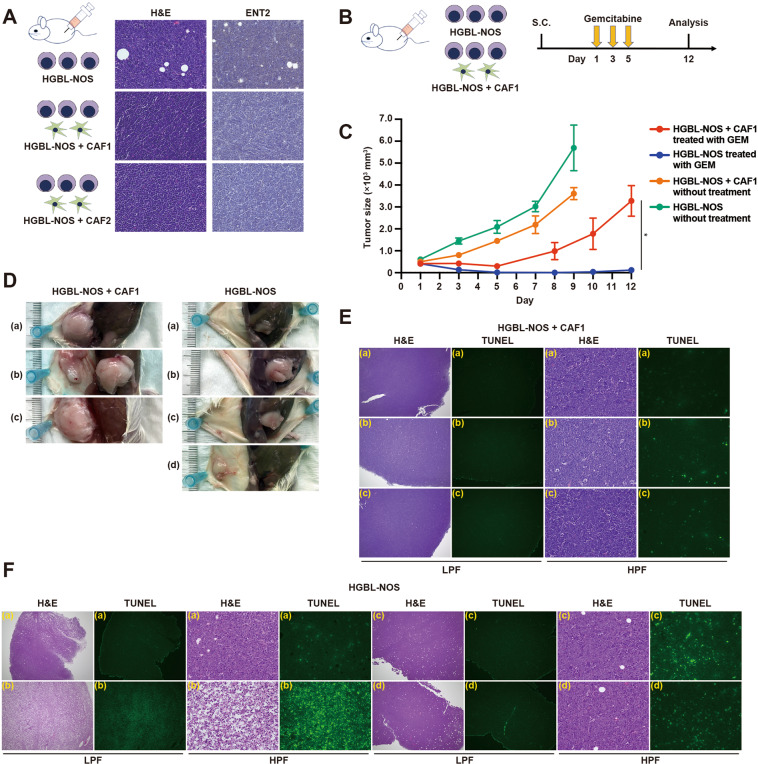
CAFs affect ENT2 expression and anti-pyrimidine susceptibility in xenograft models. **A** ENT2 expression in HGBL-NOS cells with or without CAFs subcutaneously transplanted in the flanks of the xenograft model. Pathological specimens of tumors from HGBL-NOS cells (upper), HGBL-NOS cells with CAF1 (center), and HGBL-NOS cells with CAF2 (lower) were stained with hematoxylin and eosin (left) and ENT2 (right). **B** Schema of in vivo experiments. After tumors formed up to 300 mm^3^ from HGBL-NOS cells or HGBL-NOS cells with CAF1, mice were treated with gemcitabine (*n* = 3 for HGBL-NOS cells with CAF1, *n* = 4 for HGBL-NOS cells) for 3 days. Untreated mice (*n* = 4 for HGBL-NOS cells with CAF1, *n* = 4 for HGBL-NOS cells) were set as controls. Tumor volumes were measured, and mice treated with gemcitabine were killed and analyzed on day 12. Mice untreated were killed on day 9 when the tumor size exceeded 2500 mm^3^. **C** Tumor volumes from HGBL-NOS cells with CAF1 (red line) and HGBL-NOS cells (blue line) treated with gemcitabine, and untreated HGBL-NOS cells with CAF1 (orange line) and HGBL-NOS cells (green line). Each point is the mean taken from 3 or 4 mice with error bars indicating standard error. Asterisk indicates *p* value: **0.01 > *p* ≥ 0.001. **D** Photographs of mice injected with HGBL-NOS cells with CAF1 (left) and HGBL-NOS cells (right) after gemcitabine treatment on day 12. **E** Pathological specimens of 3 tumors [(a) to (c)] from HGBL-NOS cells with CAF1 on day 12. Specimens stained with H&E (left, low power field [LPF]; center right, high power field [HPF]) and TUNEL (center left; LPF, right; HPF) are shown. **F** Pathological specimens of 4 tumors [(a) to (d)] from HGBL-NOS cells on day 12. Specimens of each tumor stained with H&E (left, LPF; center right, HPF) and TUNEL (center left, LPF; right; HPF) are shown. Original magnifications: LPF ×40 and HPF ×400.

Subsequently, the resistance to gemcitabine was confirmed by in vivo experiments. First, we subcutaneously inoculated lymphoma cells with or without CAFs in the lower flanks of NOG mice. In tumors formed by lymphoma cells alone, ENT2 was expressed according to IHC staining, while ENT2 expression was suppressed in tumors formed by a mixture of lymphoma cells and CAFs (Figs. 8A and S7A). Next, we compared the chemosensitivity to gemcitabine in tumors formed by lymphoma cells alone with those by a mixture of lymphoma cells and CAFs. After intraperitoneal treatment with gemcitabine (Figs. 8B and S7B), tumors formed with CAFs displayed resistance, while it was highly effective in tumors formed by lymphoma cells alone (Figs. 8C, D, S7C, D). In tumor tissues after gemcitabine treatment, lymphoma cell growth was retained and almost all cells were alive in tumors formed by lymphoma cells with CAFs (Fig. 8E), while significant cell death was observed in parts of tumors formed by lymphoma cells alone (Fig. 8F).

### ENT2 expression in clinical practice

Finally, the association of ENT2 expression with chemosensitivity to gemcitabine was examined using pathological specimens (Table S6). The use of gemcitabine is approved by the Japanese insurance system in patients with relapsed or refractory malignant lymphoma. We thus studied pathological specimens obtained from DLBCL patients just before receiving gemcitabine, including the GCDR regimen. In those who responded to GCDR, the cytoplasmic expression of ENT2 was observed in pathological specimens, while ENT2 expression was at least in part not observed in non-responders (Fig. S8). These data suggested that ENT2 expression might affect chemosensitivity to gemcitabine in clinical practice.

## Discussion

In the present study, we found that exosomes secreted from CAFs were involved in the survival of lymphoma cells through the enhancement of glycolysis. We further demonstrated that miR-4717-5p, which was translocated from CAFs to tumor cells via exosomes, induced anti-pyrimidine resistance by modulating the pyrimidine transporter ENT2. In addition, the expression of ENT2 was significantly clinically related to the susceptibility to gemcitabine, including in the GCDR regimen. In in vivo experiments, CAFs were found to be involved in the resistance to gemcitabine. Our data suggest that exosomes from CAFs have clinical significance and are a potential therapeutic target.

In this study, we found that exosomes from CAFs, in addition to other factors such as metabolites that we discovered earlier, were involved in supporting lymphoma cells. CAFs were isolated from primary lymph node samples of various types of lymphomas, and the extent of support differed among CAFs, which was at least in part due to the amount of exosome secretion. Considering that plenty of pyruvate is secreted from CAFs according to our previous report [15], exosomes might play a pivotal role in the variable survival of lymphoma cells.

We attempted to uncover the clinical significance of CAFs and their derived exosomes. In the presence of co-culture with CAFs, the induction of resistance to gemcitabine, cytarabine, and bendamustine was observed, though resistance to bendamustine was not induced in the presence of exosomes from CAFs. Considering that the uptake of bendamustine into lymphoma cells is thought to be through human organic cation transporter 1 (OCT1) and human organic anion transporter 3 (OAT3) [29, 30], the difference in drug transporters between anti-pyrimidine drugs and bendamustine might have caused this discrepancy. In addition, our finding that resistance to bendamustine was not observed in the presence of exosomes suggests that drug resistance by CAFs is induced by varying mechanisms. Our finding that miR-4717-5p did not change *SLC29A2* mRNA expression, but rather decreased the expression of its encoded protein, leads us to speculate that miR-4717-5p does not directly target *SLC29A2*, but may reverse the suppression of ENT2 degradation by inducing decreased deubiquitinase.

The present study showed that the expression of ENT2 in pathological tissues might be associated with susceptibility to gemcitabine. Currently, gemcitabine is a key drug in the treatment of lymphoma, in particular in salvage treatment [26, 31]. There is no established protocol to select salvage regimens; the immunohistochemical staining of ENT2 in pathological specimens might contribute to the optimal choice of salvage treatment in patients with relapsed or refractory disease. Moreover, the knockdown of ENT2 expression using a specific siRNA partially restored resistance to gemcitabine. In in vivo experiments, tumors that were grown simultaneously with lymphoma cells and CAFs demonstrated resistance to gemcitabine treatment. These findings suggest that CAFs and their derived exosomes are clinically relevant and that exosomes secreted from CAFs are a potential therapeutic target.

Although the clinical significance of exosomes secreted from tumor cells has been investigated previously [16, 28, 32], we demonstrated that exosomes secreted from CAFs were involved in the survival of lymphoma cells and in drug resistance to anti-pyrimidine drugs in the present study. In addition, our results suggest that CAF-derived exosomes have clinical significance through the modulation of ENT2 expression. Although the present findings are novel and provide useful information on the role of CAFs, our findings were based on a small number of patient samples. Obvious limitations exist, and future validation is warranted to uncover the role of CAFs and their derived exosomes in the lymphoma microenvironment.

## Supplementary information

Supplemental information

Table S1

Table S2

Table S3

Table S4

Table S5

Table S6

Figure S1

Figure S2

Figure S3

Figure S4

Figure S5

Figure S6

Figure S7

Figure S8

## References

[1] Coiffier B, Thieblemont C, Van Den Neste E, Lepeu G, Plantier I, Castaigne S (2010). Long-term outcome of patients in the LNH-98.5 trial, the first randomized study comparing rituximab-CHOP to standard CHOP chemotherapy in DLBCL patients: a study by the Groupe d’Etudes des Lymphomes de l’Adulte. Blood.

[2] Neelapu SS, Locke FL, Bartlett NL, Lekakis LJ, Miklos DB, Jacobson CA (2017). Axicabtagene ciloleucel CAR T-cell therapy in refractory large B-cell lymphoma. N. Engl J Med.

[3] Ishida T, Joh T, Uike N, Yamamoto K, Utsunomiya A, Yoshida S (2012). Defucosylated anti-CCR4 monoclonal antibody (KW-0761) for relapsed adult T-cell leukemia-lymphoma: a multicenter phase II study. J Clin Oncol.

[4] Horwitz S, O’Connor OA, Pro B, Illidge T, Fanale M, Advani R (2019). Brentuximab vedotin with chemotherapy for CD30-positive peripheral T-cell lymphoma (ECHELON-2): a global, double-blind, randomised, phase 3 trial. Lancet.

[5] Armand P, Shipp MA, Ribrag V, Michot JM, Zinzani PL, Kuruvilla J (2016). Programmed death-1 blockade with pembrolizumab in patients with classical Hodgkin lymphoma after brentuximab vedotin failure. J Clin Oncol.

[6] Crump M, Neelapu SS, Farooq U, Van Den Neste E, Kuruvilla J, Westin J (2017). Outcomes in refractory diffuse large B-cell lymphoma: results from the international SCHOLAR-1 study. Blood.

[7] Smith A, Crouch S, Lax S, Li J, Painter D, Howell D (2015). Lymphoma incidence, survival and prevalence 2004-14: sub-type analyses from the UK’s Haematological Malignancy Research Network. Br J cancer.

[8] Dave SS, Wright G, Tan B, Rosenwald A, Gascoyne RD, Chan WC (2004). Prediction of survival in follicular lymphoma based on molecular features of tumor-infiltrating immune cells. N. Engl J Med.

[9] Azmi AS, Bao B, Sarkar FH (2013). Exosomes in cancer development, metastasis, and drug resistance: a comprehensive review. Cancer Metastasis Rev.

[10] Chen WJ, Ho CC, Chang YL, Chen HY, Lin CA, Ling TY (2014). Cancer-associated fibroblasts regulate the plasticity of lung cancer stemness via paracrine signalling. Nat Commun.

[11] Taskinen M, Karjalainen-Lindsberg ML, Nyman H, Eerola LM, Leppa S (2007). A high tumor-associated macrophage content predicts favorable outcome in follicular lymphoma patients treated with rituximab and cyclophosphamide-doxorubicin-vincristine-prednisone. Clin Cancer Res.

[12] An J, Enomoto A, Weng L, Kato T, Iwakoshi A, Ushida K (2013). Significance of cancer-associated fibroblasts in the regulation of gene expression in the leading cells of invasive lung cancer. J Cancer Res Clin Oncol.

[13] Orimo A, Gupta PB, Sgroi DC, Arenzana-Seisdedos F, Delaunay T, Naeem R (2005). Stromal fibroblasts present in invasive human breast carcinomas promote tumor growth and angiogenesis through elevated SDF-1/CXCL12 secretion. Cell.

[14] Aoki T, Shimada K, Sakamoto A, Sugimoto K, Morishita T, Kojima Y (2017). Emetine elicits apoptosis of intractable B-cell lymphoma cells with MYC rearrangement through inhibition of glycolytic metabolism. Oncotarget.

[15] Sakamoto A, Kunou S, Shimada K, Tsunoda M, Aoki T, Iriyama C (2019). Pyruvate secreted from patient-derived cancer-associated fibroblasts supports survival of primary lymphoma cells. Cancer Sci.

[16] Skog J, Wurdinger T, van Rijn S, Meijer DH, Gainche L, Sena-Esteves M (2008). Glioblastoma microvesicles transport RNA and proteins that promote tumour growth and provide diagnostic biomarkers. Nat Cell Biol.

[17] Svensson KJ, Kucharzewska P, Christianson HC, Skold S, Lofstedt T, Johansson MC (2011). Hypoxia triggers a proangiogenic pathway involving cancer cell microvesicles and PAR-2-mediated heparin-binding EGF signaling in endothelial cells. Proc Natl Acad Sci USA.

[18] Taylor DD, Gercel-Taylor C, Lyons KS, Stanson J, Whiteside TL (2003). T-cell apoptosis and suppression of T-cell receptor/CD3-zeta by Fas ligand-containing membrane vesicles shed from ovarian tumors. Clin Cancer Res.

[19] Luga V, Zhang L, Viloria-Petit AM, Ogunjimi AA, Inanlou MR, Chiu E (2012). Exosomes mediate stromal mobilization of autocrine Wnt-PCP signaling in breast cancer cell migration. Cell.

[20] Richards KE, Zeleniak AE, Fishel ML, Wu J, Littlepage LE, Hill R (2017). Cancer-associated fibroblast exosomes regulate survival and proliferation of pancreatic cancer cells. Oncogene.

[21] Shimada K, Shimada S, Sugimoto K, Nakatochi M, Suguro M, Hirakawa A (2016). Development and analysis of patient-derived xenograft mouse models in intravascular large B-cell lymphoma. Leukemia.

[22] Sugimoto K, Hayakawa F, Shimada S, Morishita T, Shimada K, Katakai T (2015). Discovery of a drug targeting microenvironmental support for lymphoma cells by screening using patient-derived xenograft cells. Sci Rep.

[23] Shimada K, Tomita A, Minami Y, Abe A, Hind CK, Kiyoi H (2012). CML cells expressing the TEL/MDS1/EVI1 fusion are resistant to imatinib-induced apoptosis through inhibition of BAD, but are resensitized with ABT-737. Exp Hematol.

[24] Takagi Y, Shimada K, Shimada S, Sakamoto A, Naoe T, Nakamura S (2016). SPIB is a novel prognostic factor in diffuse large B-cell lymphoma that mediates apoptosis via the PI3K-AKT pathway. Cancer Sci.

[25] Ostrowski M, Carmo NB, Krumeich S, Fanget I, Raposo G, Savina A (2010). Rab27a and Rab27b control different steps of the exosome secretion pathway. Nat Cell Biol.

[26] Gopal AK, Press OW, Shustov AR, Petersdorf SH, Gooley TA, Daniels JT (2010). Efficacy and safety of gemcitabine, carboplatin, dexamethasone, and rituximab in patients with relapsed/refractory lymphoma: a prospective multi-center phase II study by the Puget Sound Oncology Consortium. Leuk Lymphoma.

[27] Cheson BD, Pfistner B, Juweid ME, Gascoyne RD, Specht L, Horning SJ (2007). Revised response criteria for malignant lymphoma. J Clin Oncol.

[28] Kosaka N, Iguchi H, Hagiwara K, Yoshioka Y, Takeshita F, Ochiya T (2013). Neutral sphingomyelinase 2 (nSMase2)-dependent exosomal transfer of angiogenic microRNAs regulate cancer cell metastasis. The. J Biol Chem.

[29] Arimany-Nardi C, Montraveta A, Lee-Verges E, Puente XS, Koepsell H, Campo E (2015). Human organic cation transporter 1 (hOCT1) as a mediator of bendamustine uptake and cytotoxicity in chronic lymphocytic leukemia (CLL) cells. Pharmacogenomics J.

[30] Hagos Y, Hundertmark P, Shnitsar V, Marada VV, Wulf G, Burckhardt G (2015). Renal human organic anion transporter 3 increases the susceptibility of lymphoma cells to bendamustine uptake. Am J Physiol Ren Physiol.

[31] Crump M, Baetz T, Couban S, Belch A, Marcellus D, Howson-Jan K (2004). Gemcitabine, dexamethasone, and cisplatin in patients with recurrent or refractory aggressive histology B-cell non-Hodgkin lymphoma: a Phase II study by the National Cancer Institute of Canada Clinical Trials Group (NCIC-CTG). Cancer.

[32] Peinado H, Aleckovic M, Lavotshkin S, Matei I, Costa-Silva B, Moreno-Bueno G (2012). Melanoma exosomes educate bone marrow progenitor cells toward a pro-metastatic phenotype through MET. Nat Med.

