# Supplementary material for: Exosomes secreted from cancer-associated fibroblasts elicit anti-pyrimidine drug resistance through modulation of its transporter in malignant lymphoma
Source: Oncogene. 2021 May 16;40(23):3989–4003. doi: 10.1038/s41388-021-01829-y (PMC8195743; doi:10.1038/s41388-021-01829-y)
Supplement: Supplementary file 1 — Supplemental information [file 41388_2021_1829_MOESM1_ESM.docx]

**Supplemental information**

**Supplemental Methods**

**Tumor cells from patient-derived xenograft models**

NOD/Shi-*scid* IL2Rγ^null^ (NOG) mice were purchased from In-Vivo Science Inc., Tokyo, Japan. To develop the PDX mouse model, 1.0 × 10^6^ ~ 5.0 × 10^6^ primary lymph node cells or bone marrow mononuclear cells from patients with malignant lymphoma were transplanted intravenously, intraperitoneally, or subcutaneously into NOG mice. To prevent the proliferation of human T cells in NOG mice, 100 μg of OKT3, an anti-CD3 monoclonal antibody (BioLegend, San Diego, CA, USA), was also injected intraperitoneally on the same day. The engraftment of B-cell lymphoma cells was confirmed via the expression of human CD45 (BD, Franklin Lakes, NJ, USA or BioLegend) and human CD19 (BioLegend) using flow cytometry (FCM) (FACSCalibur or FACSCanto^TM^ II, BD) and pathological specimens, and then tumor cells were cryopreserved until used in experiments. All animal experimental procedures complied with the Regulations on Animal Experiments of Nagoya University.

**Establishment of CAFs**

CAFs were established as described previously [1, 3]. In brief, patient lymph node samples were mashed to obtain a cell suspension for subsequent diagnostic analyses. The residue was cultured in Iscove's Modified Dulbecco's Medium (IMDM) (Sigma-Aldrich, St. Louis, MO, USA) supplemented with 10% fetal bovine serum (FBS) (GIBCO, Thermo Fisher Scientific, Waltham, MA, USA) or 10% human serum provided by healthy volunteers. The cell culture medium contained penicillin/streptomycin and 2 mM L-glutamine. Of the various types of cells in this culture, only the spindle-shaped adherent cells that were α-smooth muscle actin (SMA) positive and that survived and proliferated for more than several months were isolated [2]. Since such adherent cells were not established from benign disease samples, these patient-derived adherent cells were regarded as CAFs. These CAFs were maintained in the above-mentioned culture conditions by splitting them once or twice a week.

**Cell line culture**

SU-DHL4 and SU-DHL6 were cultured in RPMI 1640 (GIBCO) supplemented with 10% FBS, 2 mM L-glutamine, 100 U/mL penicillin, and 100 μg/mL streptomycin. OCI-Ly10 was cultured in IMDM (GIBCO) supplemented with 10% FBS, 2 mM L-glutamine, 100 U/mL penicillin, and 100 μg/mL streptomycin. OCI-Ly3 was cultured in IMDM (GIBCO) supplemented with 20% FBS 2 mM L-glutamine, 100 U/mL penicillin, and 100 μg/mL streptomycin.

**Cell viability and cytotoxicity assessment**

To assess the cell viability and cytotoxicity of lymphoma cells co-cultured with CAFs, we evaluated them using an image analyzer as described previously [1, 5]. In brief, 2.5 × 10^3^ CAFs were placed in 96-well plates and incubated for 24 h. Subsequently, 1 × 10^4^ lymphoma cells were added into each well and then co-cultured with the CAFs. For anticancer drug assessment, an appropriate anticancer drug was then added into each well. After a 48-h incubation, total and dead cells were stained with Hoechst 33342 (Invitrogen, Thermo Fisher Scientific, Carlsbad, CA, USA) and 15 μg/ml of propidium iodide (PI). Dead lymphoma cells were selectively counted with an Array Scan VTI HCS Reader (Thermo Fisher Scientific).

To evaluate the death of lymphoma cells in the presence of exosomes derived from CAFs, WST-8 assays were performed. Ten thousand lymphoma cells were seeded with 5 × 10^9^ exosomes in 96-well plates and incubated for 72 h. For anticancer drug assessment, an appropriate anticancer drug was added into each well at the same time. After incubation, 10 μl of the Cell Counting Kit-8 reagent (Dojindo Laboratory, Kumamoto, Japan) was added into each well and incubated for 2 h, and then fluorescence was evaluated at 450 nM using the GloMax®-Multi Detection System (Promega, Madison, WI, USA).

To evaluate tumor cell death, a PI or 7-AAD, and Annexin V-fluorescein isothiocyanate (FITC) assay was performed as described in detail previously [4, 6]. In brief, cells were seeded at a concentration of 1 × 10^5^ cells/ml with exosomes at a concentration of 5 × 10^10^ particles/ml in 6-well plates or 12-well plates. The mixtures were incubated with appropriate anticancer agents for 72 h, then stained with 10 μg/ml of PI or 0.5 ng/μl 7-AAD, and 10 μg/ml of Annexin V-FITC for 15 min at room temperature in the dark. Cell death was assessed using FCM (BD) and analyzed using FlowJo Version 10 software (TreeStar, Ashland, OR, USA).

To analyze the survival of lymphoma cell lines in the presence of CAF-derived exosomes, ten thousand lymphoma cells were seeded with 0.8 × 10^9^ exosomes and an appropriate anti-cancer drug in 96-well plates and incubated for 48 h. Then CellTiter-Glo 3D Reagent was added into each well and incubated for 30 min according to the manufacturer’s protocol, and then luminescence was evaluated using the GloMax®-Multi Detection System (Promega).

To evaluate the effects of miRNAs on *SLC29A2* expression and survival in lymphoma cells, lymphoma cells were transfected with 100 nM of miRNA using Lipofectamine RNAiMAX reagent (Invitrogen) according to the manufacturer’s protocol. MiR-1915-3p (5’-CCCC AGGG CGAC GCGG CGGG-3’), miR-4715-3p (5’-ACAC AUGG GUGG CUGU GGCC U-3’), and miR-4715-5p (5’-UAGG CCAC AGCC ACCC AUGU GU-3’) were obtained from Ajinomoto Bio-Pharma (Osaka, Japan). Negative control miRNA was purchased from Bioneer (Daejeon, Korea). Forty-five thousand lymphoma cells were placed with appropriate anti-cancer drugs in 96-well plates and incubated for 48 h. Then survival of lymphoma cells was evaluated using CellTiterGlo3D (Promega).

**Quantitative real-time reverse transcriptase (RT)-PCR**

RNA was extracted from cell lysates (QIAmp RNA Blood Mini Kit, Qiagen), and complementary DNA was prepared with a PrimeScript^TM^ II 1^st^ strand cDNA Synthesis Kit (Takara Bio, Kusatsu, Japan) according to the manufacturer’s protocol. Quantitative RT-PCR analysis of the expression of *SLC29A2* (Assay ID: Hs02513021_s1, Applied Biosystems in Thermo Fisher Scientific, Foster City, CA, USA) was carried out with Universal PCR Master Mix, No AmpErase® UNG using an Applied Biosystems 7300 Real-Time PCR system (Applied Biosystems). Data were normalized by the amount of eukaryotic 18S rRNA endogenous control using gene-specific primers (Assay ID: Hs99999901_s1, Applied Biosystems).

**Supplemental Reference**

1 Aoki T, Shimada K, Sakamoto A, Sugimoto K, Morishita T, Kojima Y *et al*. Emetine elicits apoptosis of intractable B-cell lymphoma cells with MYC rearrangement through inhibition of glycolytic metabolism. Oncotarget 2017; 8: 13085-13098.

2 Rasanen K, Vaheri A. Activation of fibroblasts in cancer stroma. Exp Cell Res 2010; 316: 2713-2722.

3 Sakamoto A, Kunou S, Shimada K, Tsunoda M, Aoki T, Iriyama C *et al*. Pyruvate secreted from patient-derived cancer-associated fibroblasts supports survival of primary lymphoma cells. Cancer Sci 2019; 110: 269-278.

4 Shimada K, Tomita A, Minami Y, Abe A, Hind CK, Kiyoi H *et al*. CML cells expressing the TEL/MDS1/EVI1 fusion are resistant to imatinib-induced apoptosis through inhibition of BAD, but are resensitized with ABT-737. Experimental hematology 2012; 40: 724-737 e722.

5 Sugimoto K, Hayakawa F, Shimada S, Morishita T, Shimada K, Katakai T *et al*. Discovery of a drug targeting microenvironmental support for lymphoma cells by screening using patient-derived xenograft cells. Scientific Reports 2015; 5: 13054.

6 Takagi Y, Shimada K, Shimada S, Sakamoto A, Naoe T, Nakamura S *et al*. SPIB is a novel prognostic factor in diffuse large B-cell lymphoma that mediates apoptosis via the PI3K-AKT pathway. Cancer Sci 2016; 107: 1270-1280.

**Figure legends for supplemental figures**

**Figure S1. Viability of tumor cells with various CAFs.**

**(A to E)** Viability of various lymphoma cell types from PDX models [Lymphoblastic lymphoma **(A)**, DLBCL **(B)**] or primary lymphoma cells [FL1 **(C)**, FL2 **(D)**, and PTCL-NOS **(E)**] in monoculture and co-culture with CAF1 to CAF4.

**Figure S2. Survival of lymphoma cells in the presence of exosomes from CAFs**

**(A)** Uptake of exosomes from CAFs into lymphoma cells. Fluorescent microscopic images of exosomes stained with PKH26 (red), tumor cells stained with Hoechst 33342 (blue), and merged images are shown. **(a)** HGBL-NOS cells and exosomes from CAF2, **(b)** HGBL-NOS cells and exosomes from CAF3, **(c)** HGBL-NOS cells and exosomes from CAF4, **(d)** HGBL-NOS cells only, **(e)** BL cells and exosomes from CAF1, **(f)** BL cells and exosomes from CAF2, **(g)** BL cells and exosomes from CAF3, **(h)** BL cells and exosomes from CAF4, and **(i)** BL cells only are shown. **(B)** Immunoblotting for HK2, PDK1, and tubulin as a loading control in BL cells in monoculture and in the presence of exosomes from CAF1 to CAF4. **(C)** Viability of HGBL-NOS cells in the presence of various concentrations of exosomes from CAF2. Ns, not significant.

**Figure S3. Metabolites of lymphoma cells in the presence of CAF or CAF-derived exosomes**

Metabolites of glycolysis in lymphoma cells in the presence of CAF1-derived exosomes or co-cultured with CAF1 analyzed with CE-TOFMS and CE-QqQMS are shown.

**Figure S4.** **Change of susceptibility to anti-pyrimidine drugs induced by CAFs and their derived exosomes**

**(A)** Viability of BL cells in monoculture and co-culture with CAF1 to CAF4 in the presence of 200 nM gemcitabine. **(B)** Assessment of cell death of BL cells treated with 200 nM gemcitabine in co-culture with CAF1 to CAF4. **(C)** Relative viability of BL cells in the presence of exosomes from CAF1 and CAF2. **(D)** Assessment of cell death of BL cells treated with 200 nM gemcitabine in the presence of exosomes from CAF1 and CAF2. **(E)** Cell death of HGBL-NOS cells co-cultured with CAF2 transfected with siRNA for RAB27B. **(F)** Cell death of BL cells co-cultured with CAF1 transfected with siRNA for RAB27B. **(G)** Cell death of BL cells co-cultured with CAF2 transfected with siRNA for RAB27B. Asterisks indicate *p* values as follows: ^*^ 0.05 > *p* ≥ 0.01, ^**^ 0.01 > *p* ≥ 0.001, ^****^ 0.0001 > *p*. Each bar is the mean value from 3 or more independent experiments with error bars indicating standard error. Ns, not significant.

**Figure S5. Susceptibility to anti-cancer drugs induced by CAFs and their derived exosomes**

**(A)** Viability of BL cells in monoculture and co-culture with CAF1 to CAF4 in the presence of 100 nM cytarabine. **(B)** Viability of HGBL-NOS cells in monoculture and co-culture with CAF1 to CAF4 in the presence of 25 μM bendamustine. **(C)** Relative viability of BL cells treated with 100 nM cytarabine in the presence of exosomes from CAF1 and CAF2. **(D)** Viability of representative FL cell lines and DLBCL cell lines in monoculture and co-culture with CAF1 and CAF4. Data were taken from a single experiment. Asterisks indicate *p* values as follows: ^*^ 0.05 > *p* ≥ 0.01, ^**^ 0.01 > *p* ≥ 0.001, ^***^ 0.001 > *p* ≥ 0.0001. Each bar is the mean taken from three or more independent experiments with error bars indicating standard error, except for (D).

**Figure S6. Expression of an anti-pyrimidine drug transporter and intracellular concentrations of the drugs**

**(A)** Immunoblotting for ENT2 and tubulin as a loading control in BL cells in monoculture and co-culture with each CAF. **(B)** Immunoblotting for ENT2 and tubulin as a loading control in BL cells in the presence of exosomes from CAF1 and CAF2. **(C and D)** Intracellular concentrations of GEM-TP **(C)** and Ara-CTP **(D)**. BL cells in the presence of 200 nM gemcitabine **(C)** and 200 nM cytarabine **(D)** in monoculture and co-culture with CAF1 and CAF2 were evaluated. **(E and F)** Intracellular concentrations of gemcitabine **(E)** and cytarabine **(F)** in BL cells in the presence of exosomes from CAF1 and CAF2. **(G)** Relative expression of *SLC29A2* mRNA of HGBL-NOS cells transfected with each miRNA. Asterisks indicate *p* values as follows: ^*^ 0.05 > *p* ≥ 0.01, ^**^ 0.01 > *p* ≥ 0.001, ^***^ 0.001 > *p* ≥ 0.0001, ^****^ 0.0001 > *p*. Each bar is the mean taken from 3 or more independent experiments with error bars indicating standard error. Ns, not significant.

**Figure S7. CAFs affect ENT2 expression and anti-pyrimidine susceptibility in xenograft models.**

**(A)** ENT2 expression in BL cells with or without CAFs subcutaneously injected in the flanks of the xenograft model. Pathological specimens of tumors from BL cells (upper), BL cells with CAF1 (center), and BL cells with CAF2 (lower) were stained with hematoxylin and eosin (left) and ENT2 (right). **(B)** Schema of *in vivo* experiments. After tumors formed up to 300 mm^3^ from BL cells or BL cells with CAF1, mice were treated with gemcitabine (n=3 for BL cells with CAF1, n=3 for BL cells) for 3 days. Tumor volumes were measured, and mice were killed and analyzed on day 14. **(C)** Tumor volumes from BL cells with CAF1 (red line) and BL cells (blue line). Each point is the mean taken from 3 mice with error bars indicating standard error. Ns, not significant. **(D)** Photographs of mice inoculated with BL cells with CAF1 (left) and BL cells (right) after gemcitabine treatment on day 14.

**Figure S8. Pathological specimens from patients with or without clinical response to anti-pyrimidine drugs.**

Pathological specimens from anti-pyrimidine non-responders (#1 to #3) and responders (#4 to #8). Specimens stained with ENT2 (left; LPF, right; HPF) are shown. The following specimens were from the same patients at different time points: #4 and #5, and #6 and #7. Original magnifications: LPF ×40 and HPF ×400.
