# Supplementary material for: Exosomes secreted from cancer-associated fibroblasts elicit anti-pyrimidine drug resistance through modulation of its transporter in malignant lymphoma
Source: Oncogene. 2021 May 16;40(23):3989–4003. doi: 10.1038/s41388-021-01829-y (PMC8195743; doi:10.1038/s41388-021-01829-y)
Supplement: Supplementary file 2 — Table S1 [file 41388_2021_1829_MOESM2_ESM.docx]

**Table S1. Characteristics of patients whose lymphoma cells were used in experiments**

|  | Age at biopsy | Sex | Biopsy site | Primary therapy | Response to primary therapy | Outcome |
| --- | --- | --- | --- | --- | --- | --- |
| HGBL-NOS | 29 | M | LN | DA-EPOCH-R | PD | Death |
| BL | 78 | M | Colon | R-THP-COP,  DA-EPOCH-R | CR | Death |
| LBL | 55 | M | BM | R-CHOP | PR | Death |
| DLBCL | 66 | M | Nasal cavity | R-CHOP+IT | PR | Alive |
| FL1 | 74 | F | LN | Rituximab | PR | Alive |
| FL2 | 58 | F | LN | GB | CR | Alive |
| PTCL-NOS | 72 | F | LN | N/A | N/A | N/A |

Abbreviations: HGBL-NOS, high grade B-cell lymphoma-not otherwise specified; DLBCL, diffuse large B-cell lymphoma; LBL, lymphoblastic lymphoma; BL, burkitt lymphoma; FL, follicular lymphoma; PTCL-NOS, peripheral T-cell lymphoma-not otherwise specified; M, male; F, female; LN, lymph node; BM, bone marrow; DA-EPOCH-R, dose adjusted etoposide, prednisolone, vincristine, cyclophosphamide, doxorubicin, and rituximab; R-THP-COP, rituximab, pilarubicin, cyclophosphamide, vincristine, and prednisolone; R-CHOP, rituximab, cyclophosphamide, doxorubicin, vincristine, and prednisolone; IT, intrathecal chemotherapy; GB, obinutuzumab and bendamustine; CR, complete response; PR, partial response; PD, progressive disease.
