# Supplementary material for: Exosomes secreted from cancer-associated fibroblasts elicit anti-pyrimidine drug resistance through modulation of its transporter in malignant lymphoma
Source: Oncogene. 2021 May 16;40(23):3989–4003. doi: 10.1038/s41388-021-01829-y (PMC8195743; doi:10.1038/s41388-021-01829-y)
Supplement: Supplementary file 3 — Table S2 [file 41388_2021_1829_MOESM3_ESM.docx]

**Table S2. Antibodies used in this study**

| Antibody | Clone | Isotype | Company | Location | |
| --- | --- | --- | --- | --- | --- |
| HK2 | C64G5 | Rabbit IgG | Cell Signaling Technology | | Danvers, MA, USA |
| PDK1 | D37A7 | Rabbit IgG | Cell Signaling Technology | | Danvers, MA, USA |
| Cleaved Caspase-3 | Asp175 | Rabbit IgG | Cell Signaling Technology | | Danvers, MA, USA |
| Α-tubulin | Ab-1 | Mouse IgG | Millipore | | Billerica, MA, USA |
| RAB27B |  | Rabbit IgG | Proteintech Group | | Chicago, IL, USA |
| N-SMase2 | G-6 | Rabbit IgG | Santa Cruz | | Dallas, TX, USA |
| ENT2 |  | Rabbit IgG | Sigma-Aldrich | | St. Louis, MO, USA |
| CD9 | EPR2949 | Rabbit IgG | Abcam | | Cambridge, UK |
| CD63 | H-193 | Rabbit IgG | Santa cruz | | Dallas, TX, USA |
| α-SMA | 1A4 | Mouse IgG2A | R&D Systems | | Minneapolis, MN, USA |
| β-Actin | AC-15 | Mouse IgG1 | Sigma-Aldrich | | St. Louis, MO, USA |
