# Supplementary material for: Exosomes secreted from cancer-associated fibroblasts elicit anti-pyrimidine drug resistance through modulation of its transporter in malignant lymphoma
Source: Oncogene. 2021 May 16;40(23):3989–4003. doi: 10.1038/s41388-021-01829-y (PMC8195743; doi:10.1038/s41388-021-01829-y)
Supplement: Supplementary file 4 — Table S3 [file 41388_2021_1829_MOESM4_ESM.docx]

**Table S3. Characteristics of patients whose primary samples were used to establish CAFs.**

|  | Age at biopsy | Sex | Diagnosis | Biopsy site |
| --- | --- | --- | --- | --- |
| CAF1 | 58 | F | FL | Lymph node |
| CAF2 | 56 | M | T-LBL | Lymph node |
| CAF3 | 29 | M | DLBCL | Lymph node |
| CAF4 | 64 | M | DLBCL | Lymph node |
| CAF5 | 59 | F | DLBCL | Breast |
| CAF6 | 49 | M | MCL | Thyroid gland |
| CAF7 | 55 | M | FL | Lymph node |
| CAF8 | 58 | F | DLBCL | Lymph node |
| CAF9 | 74 | F | AITL | Lymph node |
| CAF10 | 72 | F | PTCL-NOS | Lymph node |
| CAF11 | 66 | F | FL | Lymph node |
| CAF12 | 54 | M | DLBCL | Lymph node |

Abbreviations: CAF, cancer associated fibroblast; M, male; F, female; FL, follicular lymphoma; T-LBL, T-lymphoblastic lymphoma; DLBCL, diffuse large B-cell lymphoma; MCL, Mantle cell lymphoma; AITL, angioimmunoblastic T-cell lymphoma; PTCL-NOS, peripheral T-cell lymphoma-not otherwise specified
