# Supplementary material for: Exosomes secreted from cancer-associated fibroblasts elicit anti-pyrimidine drug resistance through modulation of its transporter in malignant lymphoma
Source: Oncogene. 2021 May 16;40(23):3989–4003. doi: 10.1038/s41388-021-01829-y (PMC8195743; doi:10.1038/s41388-021-01829-y)
Supplement: Supplementary file 5 — Table S4 [file 41388_2021_1829_MOESM5_ESM.docx]

**Table S4. Metabolites of HGBL-NOS cells**

| **Compound name** | **mono-culture** | **cultured with exosome** | **co-cultured with CAFs** |
| --- | --- | --- | --- |
| NAD+ | 104 | 107 | 137 |
| cAMP | 0.3 | 0.6 | 0.5 |
| cGMP | N.D. | N.D. | N.D. |
| NADH | 16 | 14 | 22 |
| Xanthine | 0.9 | N.D. | 14 |
| ADP-ribose | 0.4 | 3.4 | 0.8 |
| Mevalonic acid | N.D. | N.D. | N.D. |
| UDP-glucose | 80 | 43 | 114 |
| Uric acid | 3.3 | 3.0 | 15 |
| NADP+ | 5.5 | 4.1 | 5.7 |
| IMP | 78 | 91 | 186 |
| Sedoheptulose 7-phosphate | N.D. | N.D. | N.D. |
| Glucose 6-phosphate | 3.9 | 8.4 | 9.4 |
| Fructose 6-phosphate | 1.4 | 2.2 | 3.4 |
| Fructose 1-phosphate | 6.9 | 2.2 | 1.9 |
| Galactose 1-phosphate | 0.9 | 1.0 | 1.3 |
| Glucose 1-phosphate | 2.0 | 2.1 | 2.8 |
| Acetoacetyl CoA | N.D. | N.D. | 0.3 |
| Acetyl CoA | 2.1 | 1.5 | 1.9 |
| Folic acid | 0.4 | 0.4 | 0.4 |
| Ribose 5-phosphate | 2.5 | 2.6 | 1.7 |
| CoA | 1.3 | 1.4 | 2.5 |
| Ribose 1-phosphate | 1.0 | 0.6 | 0.4 |
| Ribulose 5-phosphate | 1.6 | 1.4 | 1.4 |
| Xylulose 5-phosphate | N.D. | N.D. | N.D. |
| Erythrose 4-phosphate | N.D. | N.D. | N.D. |
| HMG CoA | 1.8 | 1.1 | 0.7 |
| Glyceraldehyde 3-phosphate | 6.5 | 7.7 | 5.5 |
| NADPH | 15 | 14 | 20 |
| Malonyl CoA | 0.08 | 0.10 | 0.07 |
| Phosphocreatine | 0.9 | 1.2 | 22 |
| XMP | 0.5 | 1.4 | 13 |
| Dihydroxyacetone phosphate | 17 | 17 | 19 |
| Adenylosuccinic acid | 29 | 15 | 20 |
| Fructose 1,6-diphosphate | 36 | 41 | 35 |
| 6-Phosphogluconic acid | 1.1 | 0.4 | 0.8 |
| N-Carbamoylaspartic acid | 6.6 | 48 | 12 |
| PRPP | 0.8 | 1.2 | 1.6 |
| 2-Phosphoglyceric acid | 0.9 | 1.1 | 1.5 |
| 2,3-Diphosphoglyceric acid | 34 | 35 | 34 |
| 3-Phosphoglyceric acid | 7.3 | 8.7 | 12 |
| Phosphoenolpyruvic acid | 0.05 | N.D. | 0.7 |
| GMP | 38 | 28 | 75 |
| AMP | 179 | 134 | 296 |
| 2-Oxoisovaleric acid | 2.4 | N.D. | 6.0 |
| GDP | 55 | 46 | 79 |
| Lactic acid | 2,777 | 2,774 | 4,691 |
| ADP | 351 | 279 | 400 |
| GTP | 143 | 167 | 152 |
| Glyoxylic acid | N.D. | N.D. | N.D. |
| ATP | 800 | 1,047 | 667 |
| Glycerol 3-phosphate | 188 | 98 | 182 |
| Glycolic acid | 16 | 22 | 18 |
| Pyruvic acid | 28 | 42 | 39 |
| N-Acetylglutamic acid | 3.4 | 2.9 | 3.1 |
| 2-Hydroxyglutaric acid | 11 | 17 | 48 |
| Carbamoylphosphate | N.D. | N.D. | N.D. |
| Succinic acid | 149 | 119 | 161 |
| Malic acid | 543 | 459 | 479 |
| 2-Oxoglutaric acid | 18 | 42 | 37 |
| Fumaric acid | 127 | 104 | 104 |
| Citric acid | 120 | 380 | 516 |
| cis-Aconitic acid | 1.8 | 6.0 | 7.8 |
| Isocitric acid | 1.5 | 6.7 | 14 |
| Urea | 234 | 283 | 249 |
| Gly | 2,275 | 2,654 | 3,155 |
| M | 4.3 | 4.3 | 4.2 |
| Sarcosine | N.D. | N.D. | N.D. |
| Ala | 1,659 | 1,921 | 1,918 |
| β-Ala | 77 | 84 | 131 |
| N,N-Dimethylglycine | N.D. | N.D. | N.D. |
| γ-Aminobutyric acid | 48 | 63 | 435 |
| Choline | 59 | 34 | 88 |
| Ser | 525 | 470 | 422 |
| Carnosine | 1.5 | 2.1 | 1.9 |
| Creatinine | 7.1 | 6.3 | 8.7 |
| Pro | 857 | 1,189 | 1,757 |
| Val | 203 | 181 | 229 |
| Betaine | 1.6 | N.D. | N.D. |
| Thr | 858 | 925 | 1,095 |
| Homoserine | N.D. | N.D. | N.D. |
| Betaine aldehyde | N.D. | N.D. | N.D. |
| Cys | 1.2 | 1.7 | 3.0 |
| Hydroxyproline | 19 | 24 | 29 |
| Creatine | 10 | 9.1 | 136 |
| Ile | 156 | 135 | 154 |
| Leu | 172 | 135 | 183 |
| Asn | 203 | 226 | 234 |
| Ornithine | 12 | 41 | 82 |
| Asp | 1,121 | 1,218 | 697 |
| Homocysteine | N.D. | N.D. | N.D. |
| Adenine | 1.4 | 4.0 | 28 |
| Hypoxanthine | 102 | 110 | 148 |
| Spermidine | 4.0 | 1.4 | 2.4 |
| Gln | 233 | 99 | 83 |
| Lys | 252 | 243 | 352 |
| Glu | 6,861 | 8,266 | 7,311 |
| Met | 62 | 62 | 71 |
| Guanine | 9.4 | 10 | 30 |
| His | 44 | 42 | 53 |
| Carnitine | 31 | 17 | 8.1 |
| Phe | 83 | 78 | 92 |
| Arg | 114 | 63 | 27 |
| Citrulline | 1.8 | 20 | 31 |
| Tyr | 89 | 80 | 89 |
| S-Adenosylhomocysteine | 1.1 | 1.9 | 3.0 |
| Spermine | N.D. | N.D. | N.D. |
| Trp | 20 | 20 | 23 |
| Cystathionine | 7.7 | 11 | 14 |
| Adenosine | 1.4 | 2.4 | 3.2 |
| Inosine | 8.1 | 11 | 4.9 |
| Guanosine | 0.8 | 1.3 | 1.7 |
| Argininosuccinic acid | 24 | 42 | 65 |
| Glutathione (GSSG) | 36 | 37 | 55 |
| Glutathione (GSH) | 182 | 198 | 566 |
| S-Adenosylmethionine | 18 | 20 | 40 |
| Adenylate Energy Charge | 0.7 | 0.8 | 0.6 |
| Total Adenylate | 1,329 | 1,460 | 1,363 |
| Guanylate Energy Charge | 0.7 | 0.8 | 0.6 |
| Total Guanylate | 237 | 241 | 306 |
| GSH/GSSG | 5.1 | 5.3 | 10 |
| Total Glutathione | 253 | 272 | 675 |
| NADPH/NADP+ | 2.8 | 3.4 | 3.5 |
| NADH/NAD+ | 0.2 | 0.13 | 0.2 |
| Lactate/Pyruvate | 100 | 65 | 121 |
| Glycerol 3-phosphate/DHAP | 11 | 5.8 | 9.8 |
| Total Amino Acids | 15,789 | 18,008 | 17,948 |
| Total Essential Amino Acids | 1,850 | 1,821 | 2,252 |
| Total Non-essential Amino Acids | 13,938 | 16,187 | 15,696 |
| Total Glucogenic Amino Acids | 15,364 | 17,631 | 17,413 |
| Total Ketogenic Amino Acids | 1,631 | 1,615 | 1,989 |
| Total BCAA | 531 | 451 | 565 |
| Total Aromatic Amino Acids | 192 | 178 | 205 |
| Fischer's Ratio | 2.8 | 2.5 | 2.8 |
| Total Glu-related Amino Acids | 8,110 | 9,659 | 9,231 |
| Total Pyr-related Amino Acids | 5,338 | 5,991 | 6,616 |
| Total Acetyl CoA-related Amino Acids | 601 | 532 | 712 |
| Total Fumarate-related Amino Acids | 172 | 158 | 182 |
| Total Succinyl CoA-related Amino Acids | 421 | 378 | 453 |
| Total Oxaloacetate-related Amino Acids | 1,324 | 1,445 | 931 |
| Malate/Asp | 0.5 | 0.4 | 0.7 |
| Citrulline/Ornithine | 0.2 | 0.5 | 0.4 |
| Glu/2-Oxoglutarate | 374 | 197 | 198 |
| G6P/R5P | 1.6 | 3.3 | 5.7 |
| SAM/SAH | 16 | 11 | 13 |
| Putrescine/Spermidine | 1.1 | 3.0 | 1.8 |
|  |  |  | (pmol/10^6^ cells) |
