# Supplementary material for: Exosomes secreted from cancer-associated fibroblasts elicit anti-pyrimidine drug resistance through modulation of its transporter in malignant lymphoma
Source: Oncogene. 2021 May 16;40(23):3989–4003. doi: 10.1038/s41388-021-01829-y (PMC8195743; doi:10.1038/s41388-021-01829-y)
Supplement: Supplementary file 6 — Table S5 [file 41388_2021_1829_MOESM6_ESM.docx]

**Table S5. Top 200 miRNAs in CAF-derived exosomes**

|  | **Read count** | |
| --- | --- | --- |
| **miRNA** | **CAF1 derived exosome** | **CAF2 derived exosome** |
| hsa-miR-486-3p | 1467 | 9430 |
| hsa-miR-4717-3p | 5115 | 5538 |
| hsa-miR-4717-5p | 5115 | 5533 |
| hsa-miR-589-5p | 204 | 3108 |
| hsa-miR-198 | 1346 | 1133 |
| hsa-miR-3663-3p | 183 | 1174 |
| hsa-miR-718 | 903 | 244 |
| hsa-miR-3184-3p | 116 | 514 |
| hsa-miR-6814-3p | 141 | 484 |
| hsa-miR-103b | 212 | 403 |
| hsa-miR-8066 | 316 | 287 |
| hsa-miR-4287 | 159 | 312 |
| hsa-miR-539-5p | 177 | 213 |
| hsa-miR-8079 | 215 | 170 |
| hsa-miR-6090 | 209 | 96 |
| hsa-miR-4733-5p | 123 | 149 |
| hsa-miR-4762-5p | 148 | 103 |
| hsa-miR-6872-3p | 46 | 188 |
| hsa-miR-379-3p | 102 | 131 |
| hsa-miR-1915-3p | 65 | 156 |
| hsa-miR-20b-3p | 106 | 106 |
| hsa-miR-4782-3p | 108 | 100 |
| hsa-miR-3184-5p | 79 | 129 |
| hsa-miR-4709-5p | 26 | 178 |
| hsa-miR-3973 | 111 | 81 |
| hsa-miR-5701 | 81 | 89 |
| hsa-miR-650 | 80 | 86 |
| hsa-miR-6841-5p | 66 | 100 |
| hsa-miR-3160-5p | 54 | 100 |
| hsa-miR-24-1-5p | 69 | 64 |
| hsa-miR-3613-3p | 72 | 59 |
| hsa-miR-7848-3p | 58 | 69 |
| hsa-miR-3150b-3p | 54 | 68 |
| hsa-miR-4706 | 36 | 83 |
| hsa-miR-6078 | 70 | 40 |
| hsa-miR-4454 | 20 | 89 |
| hsa-miR-5195-5p | 49 | 59 |
| hsa-miR-4679 | 27 | 80 |
| hsa-miR-199a-3p | 44 | 49 |
| hsa-miR-199b-3p | 44 | 49 |
| hsa-miR-1303 | 16 | 74 |
| hsa-miR-504-5p | 10 | 78 |
| hsa-miR-3529-3p | 38 | 48 |
| hsa-miR-3120-5p | 11 | 74 |
| hsa-miR-6848-3p | 38 | 46 |
| hsa-miR-612 | 44 | 39 |
| hsa-miR-203b-3p | 32 | 38 |
| hsa-miR-5591-5p | 46 | 22 |
| hsa-miR-3648 | 40 | 18 |
| hsa-miR-3141 | 21 | 35 |
| hsa-miR-378e | 18 | 38 |
| hsa-miR-6755-3p | 26 | 26 |
| hsa-miR-3074-5p | 16 | 32 |
| hsa-miR-3192-3p | 10 | 38 |
| hsa-miR-4290 | 7 | 41 |
| hsa-miR-3161 | 15 | 31 |
| hsa-miR-151a-3p | 12 | 33 |
| hsa-miR-194-3p | 8 | 34 |
| hsa-miR-3653-3p | 10 | 31 |
| hsa-miR-3157-5p | 28 | 12 |
| hsa-miR-579-3p | 7 | 33 |
| hsa-miR-4743-3p | 4 | 36 |
| hsa-miR-1910-5p | 17 | 17 |
| hsa-miR-578 | 15 | 19 |
| hsa-miR-7110-3p | 13 | 21 |
| hsa-miR-765 | 12 | 22 |
| hsa-miR-6791-3p | 5 | 29 |
| hsa-miR-7-2-3p | 16 | 16 |
| hsa-miR-6810-5p | 15 | 17 |
| hsa-miR-6069 | 15 | 16 |
| hsa-miR-6776-5p | 14 | 17 |
| hsa-miR-29b-3p | 5 | 26 |
| hsa-miR-1290 | 17 | 11 |
| hsa-miR-3115 | 8 | 20 |
| hsa-miR-5195-3p | 7 | 21 |
| hsa-miR-548q | 13 | 14 |
| hsa-miR-517a-3p | 9 | 18 |
| hsa-miR-517b-3p | 9 | 18 |
| hsa-miR-6743-5p | 7 | 20 |
| hsa-miR-583 | 11 | 15 |
| hsa-miR-582-5p | 10 | 15 |
| hsa-miR-548c-3p | 4 | 21 |
| hsa-miR-127-3p | 13 | 11 |
| hsa-miR-653-3p | 11 | 13 |
| hsa-miR-6895-5p | 11 | 13 |
| hsa-miR-4697-5p | 4 | 20 |
| hsa-miR-4782-5p | 11 | 12 |
| hsa-miR-3199 | 14 | 8 |
| hsa-miR-1302 | 4 | 17 |
| hsa-miR-548ak | 1 | 20 |
| hsa-miR-6782-5p | 11 | 9 |
| hsa-miR-1273e | 9 | 11 |
| hsa-miR-4419a | 0 | 20 |
| hsa-miR-3202 | 9 | 10 |
| hsa-miR-424-5p | 5 | 14 |
| hsa-miR-548h-5p | 9 | 9 |
| hsa-miR-548ad-5p | 8 | 10 |
| hsa-miR-548ae-5p | 8 | 10 |
| hsa-miR-1911-3p | 7 | 11 |
| hsa-miR-7-5p | 5 | 13 |
| hsa-miR-4802-5p | 17 | 0 |
| hsa-miR-652-3p | 17 | 0 |
| hsa-miR-128-3p | 11 | 6 |
| hsa-miR-421 | 7 | 10 |
| hsa-miR-548v | 6 | 11 |
| hsa-miR-3591-3p | 4 | 13 |
| hsa-miR-4724-5p | 1 | 16 |
| hsa-miR-1247-3p | 13 | 3 |
| hsa-miR-4520-5p | 8 | 8 |
| hsa-miR-4676-5p | 7 | 9 |
| hsa-miR-598-3p | 5 | 11 |
| hsa-miR-671-5p | 15 | 0 |
| hsa-miR-1252-3p | 8 | 7 |
| hsa-miR-4520-2-3p | 6 | 9 |
| hsa-miR-638 | 6 | 9 |
| hsa-miR-4700-3p | 4 | 11 |
| hsa-miR-4489 | 0 | 15 |
| hsa-miR-515-3p | 0 | 15 |
| hsa-miR-502-3p | 9 | 5 |
| hsa-miR-6867-3p | 7 | 7 |
| hsa-miR-4700-5p | 4 | 10 |
| hsa-miR-450a-2-3p | 3 | 11 |
| hsa-miR-6803-5p | 3 | 11 |
| hsa-miR-1910-3p | 2 | 12 |
| hsa-miR-5590-3p | 5 | 8 |
| hsa-miR-4308 | 8 | 4 |
| hsa-miR-6766-5p | 8 | 4 |
| hsa-miR-376a-3p | 7 | 5 |
| hsa-miR-615-3p | 6 | 6 |
| hsa-miR-8063 | 6 | 6 |
| hsa-miR-877-3p | 5 | 7 |
| hsa-miR-618 | 4 | 8 |
| hsa-miR-532-3p | 1 | 11 |
| hsa-miR-4716-3p | 6 | 5 |
| hsa-miR-8055 | 6 | 5 |
| hsa-miR-4634 | 5 | 6 |
| hsa-miR-4693-3p | 4 | 7 |
| hsa-miR-1273g-3p | 3 | 8 |
| hsa-miR-4763-5p | 2 | 9 |
| hsa-miR-545-3p | 2 | 9 |
| hsa-miR-93-3p | 8 | 2 |
| hsa-miR-449b-3p | 7 | 3 |
| hsa-miR-4645-3p | 7 | 3 |
| hsa-miR-1252-5p | 6 | 4 |
| hsa-miR-1254 | 6 | 4 |
| hsa-miR-6733-3p | 5 | 5 |
| hsa-miR-3913-3p | 4 | 6 |
| hsa-miR-4693-5p | 4 | 6 |
| hsa-miR-5095 | 4 | 6 |
| hsa-miR-5688 | 4 | 6 |
| hsa-miR-127-5p | 3 | 7 |
| hsa-miR-5009-3p | 3 | 7 |
| hsa-miR-6830-3p | 3 | 7 |
| hsa-miR-4645-5p | 1 | 9 |
| hsa-miR-614 | 1 | 9 |
| hsa-miR-6805-5p | 8 | 1 |
| hsa-miR-3145-3p | 7 | 2 |
| hsa-miR-1255b-5p | 5 | 4 |
| hsa-miR-3606-5p | 5 | 4 |
| hsa-miR-508-5p | 5 | 4 |
| hsa-let-7a-3p | 4 | 5 |
| hsa-miR-23a-5p | 4 | 5 |
| hsa-miR-561-5p | 4 | 5 |
| hsa-miR-6822-3p | 4 | 5 |
| hsa-miR-145-5p | 3 | 6 |
| hsa-miR-2681-3p | 3 | 6 |
| hsa-miR-3910 | 3 | 6 |
| hsa-miR-5011-3p | 3 | 6 |
| hsa-miR-548a-3p | 3 | 6 |
| hsa-miR-203b-5p | 2 | 7 |
| hsa-miR-135a-5p | 1 | 8 |
| hsa-miR-3178 | 7 | 1 |
| hsa-miR-331-5p | 7 | 1 |
| hsa-miR-155-5p | 5 | 3 |
| hsa-miR-3163 | 5 | 3 |
| hsa-miR-3611 | 5 | 3 |
| hsa-miR-4678 | 5 | 3 |
| hsa-miR-766-3p | 5 | 3 |
| hsa-miR-3129-5p | 4 | 4 |
| hsa-miR-6509-3p | 4 | 4 |
| hsa-miR-6839-3p | 4 | 4 |
| hsa-miR-3681-3p | 3 | 5 |
| hsa-miR-3688-5p | 3 | 5 |
| hsa-miR-4769-3p | 3 | 5 |
| hsa-miR-3162-3p | 2 | 6 |
| hsa-miR-4296 | 2 | 6 |
| hsa-miR-6844 | 2 | 6 |
| hsa-miR-3198 | 0 | 8 |
| hsa-miR-4755-5p | 0 | 8 |
| hsa-miR-513b-5p | 6 | 1 |
| hsa-miR-762 | 6 | 1 |
| hsa-miR-7161-3p | 5 | 2 |
| hsa-miR-1249-3p | 4 | 3 |
| hsa-miR-3606-3p | 4 | 3 |
| hsa-miR-4472 | 4 | 3 |
| hsa-miR-507 | 4 | 3 |
| hsa-miR-548ao-5p | 4 | 3 |
| hsa-miR-6832-5p | 4 | 3 |
| hsa-miR-1183 | 3 | 4 |
| hsa-miR-1306-3p | 3 | 4 |
