# Supplementary material for: Exosomes secreted from cancer-associated fibroblasts elicit anti-pyrimidine drug resistance through modulation of its transporter in malignant lymphoma
Source: Oncogene. 2021 May 16;40(23):3989–4003. doi: 10.1038/s41388-021-01829-y (PMC8195743; doi:10.1038/s41388-021-01829-y)
Supplement: Supplementary file 7 — Table S6 [file 41388_2021_1829_MOESM7_ESM.docx]

**Table S6. Characteristics of patients whose ENT2 expression was evaluated**

| No. | Age | Sex | Biopsy site | Number of relapse | Treatment before gemcitabine | Therapeutic effect of gemcitabine |
| --- | --- | --- | --- | --- | --- | --- |
| #1 | 64 | F | LN | 2^nd^ | R-CHOP, BR | PD after 2 courses |
| #2 | 77 | M | Subcutaneous tumor | 1^st^ | R-CHOP, HD-MTX | PD after 3 courses |
| #3 | 71 | M | Intercostal tumor | 1^st^ | R-THP-COP | PD after 3 courses |
| #4^*^ | 74 | F | Subcutaneous tumor | 4^th^ | R-CHOP, BR, R-DeVIC, RT | CR after 4 courses |
| #5^*^ | 77 | F | Subcutaneous tumor | 5^th^ | R-CHOP, BR, R-DeVIC, RT, GCDR | PD after 6 courses |
| #6^†^ | 71 | M | LN | 1^st^ | R-THP-COP | PR after 5 courses |
| #7^†^ | 72 | M | Subcutaneous tumor | 2^nd^ | R-THP-COP, GCDR | PD after 6 courses |
| #8 | 70 | F | duodenum | 4^th^ | R-CHOP, R-DeVIC, R-EPOCH, CHASER | PD after 6 courses |

^*†^ The pathological specimens were obtained from the same patients at different time points.

Abbreviations: M, male; F, female; LN, lymph node; BM, bone marrow; gemcitabine; PD, progressive disease; CR, complete response; PR, Partial Response, R-CHOP, rituximab, cyclophosphamide, doxorubicin, vincristine, and prednisolone; GB, rituximab and bendamustine; HD-MTX, high dose methotrexate; R-THP-COP, rituximab, cyclophosphamide, pirarubicin, vincristine, and prednisolone; R-DeVIC, rituximab, dexamethasone, etoposide, ifosfamide, and carboplatin; RT, radiation therapy; GCDR, gemcitabine, carboplatin, dexamethasone, and rituximab; R-EPOCH, rituximab, etoposide, prednisolone, vincristine, cyclophosphamide, and doxorubicin; CHASER, cyclophosphamide, high-dose cytarabine, dexamethasone, etoposide, and rituximab.
