# Supplementary figures and images for: Exosomes secreted from cancer-associated fibroblasts elicit anti-pyrimidine drug resistance through modulation of its transporter in malignant lymphoma
Source: Oncogene. 2021 May 16;40(23):3989–4003. doi: 10.1038/s41388-021-01829-y (PMC8195743; doi:10.1038/s41388-021-01829-y)

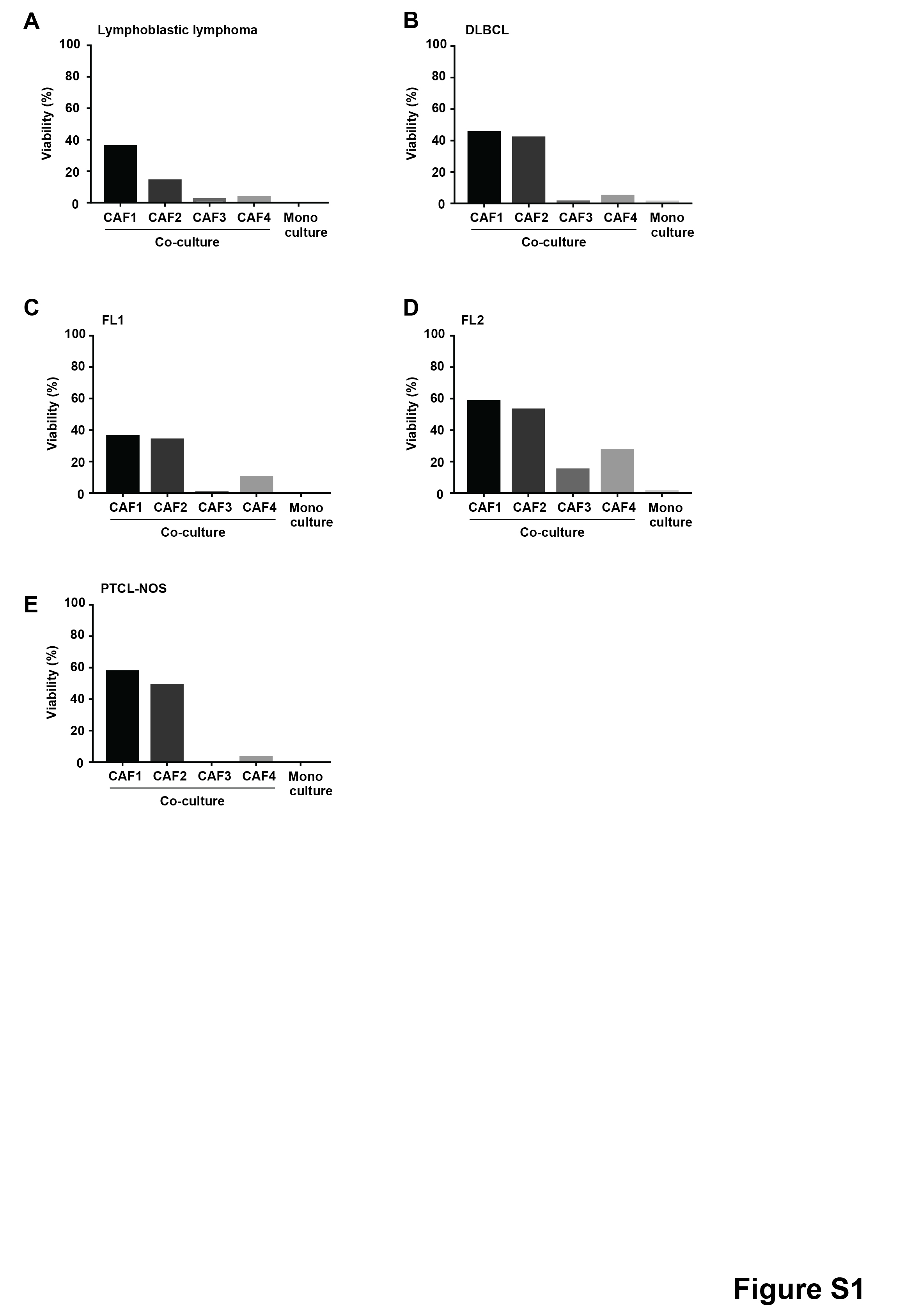

Supplement: Supplementary file 8 — Figure S1 [file 41388_2021_1829_MOESM8_ESM.jpg]

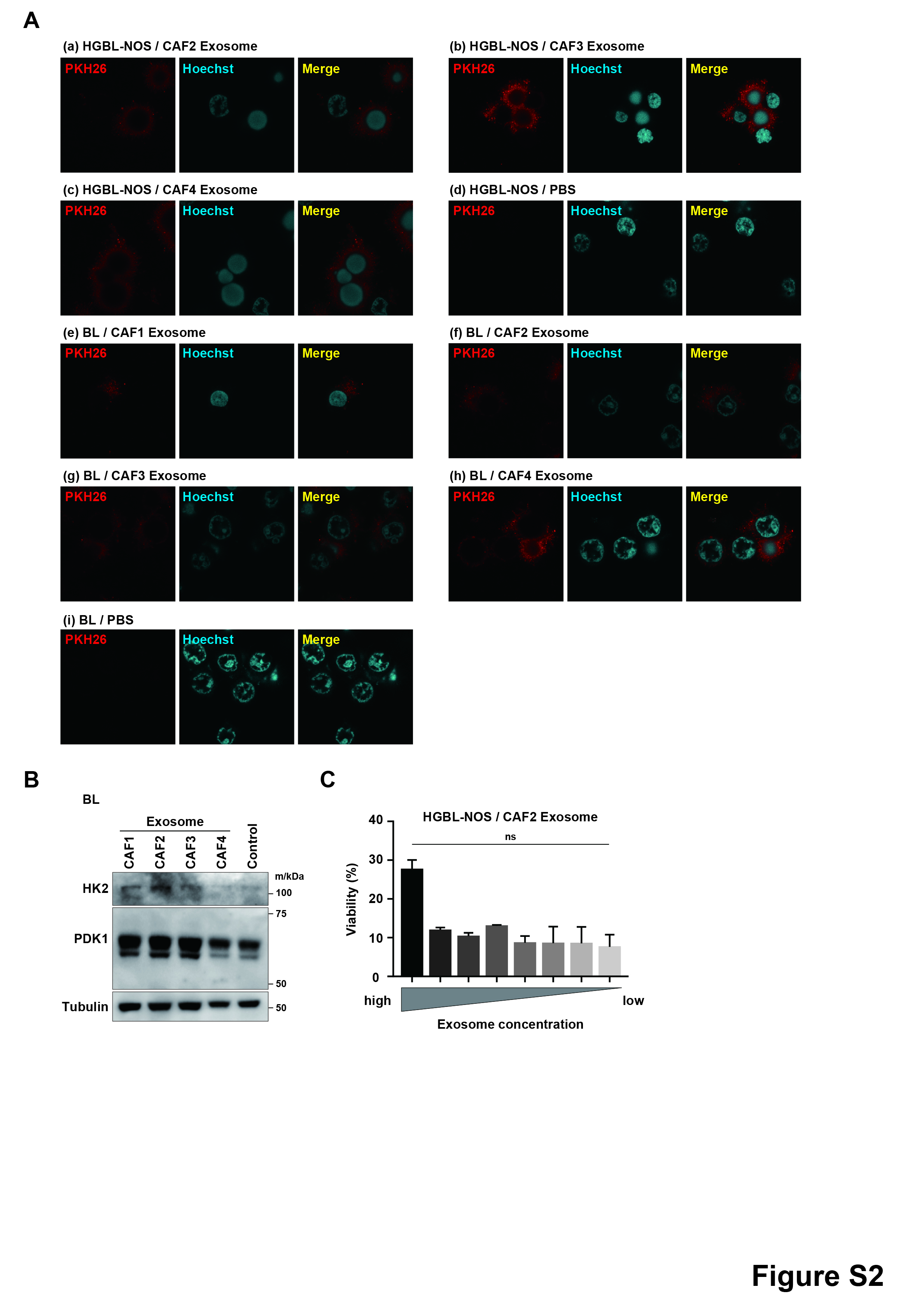

Supplement: Supplementary file 9 — Figure S2 [file 41388_2021_1829_MOESM9_ESM.jpg]

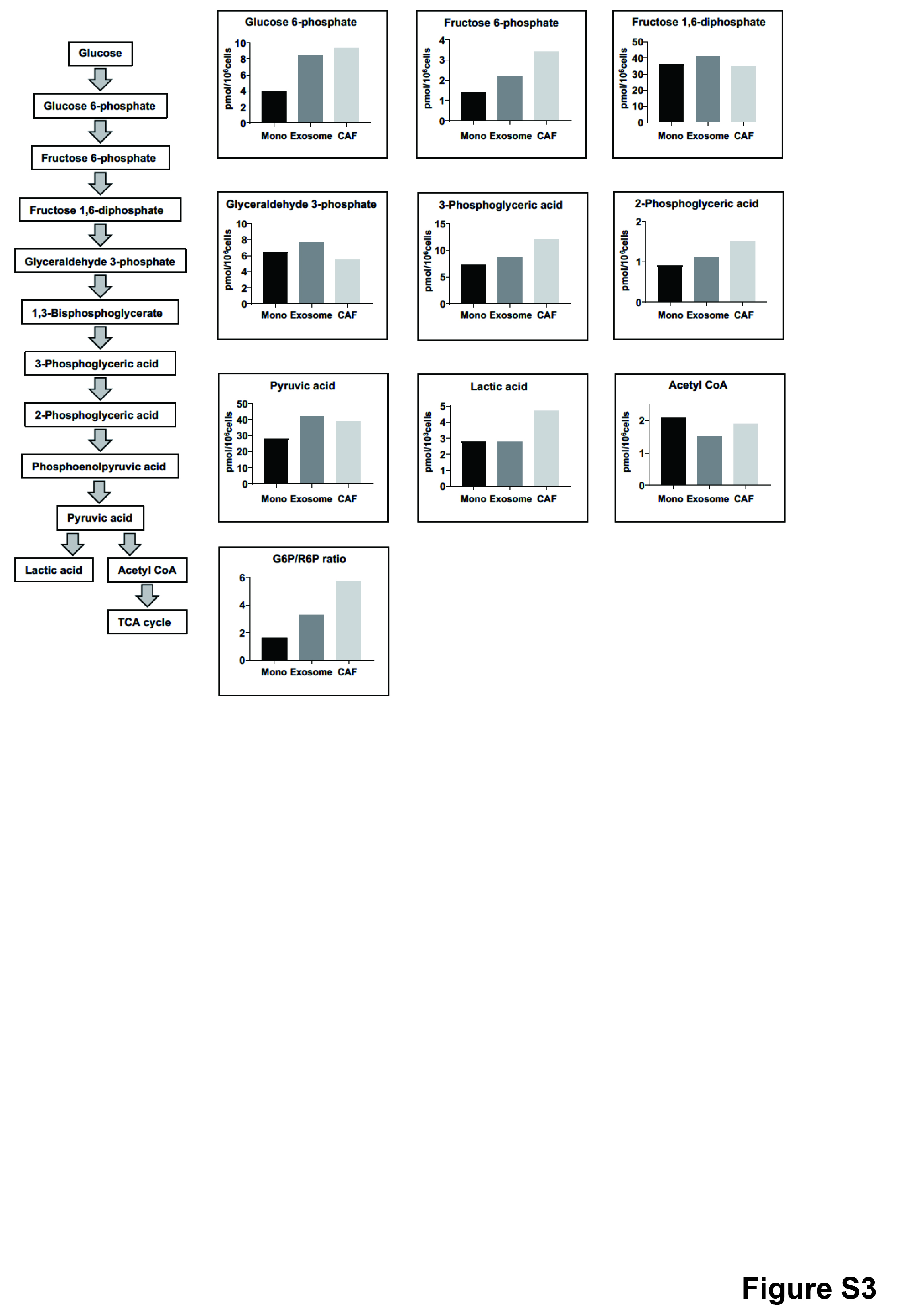

Supplement: Supplementary file 10 — Figure S3 [file 41388_2021_1829_MOESM10_ESM.jpg]

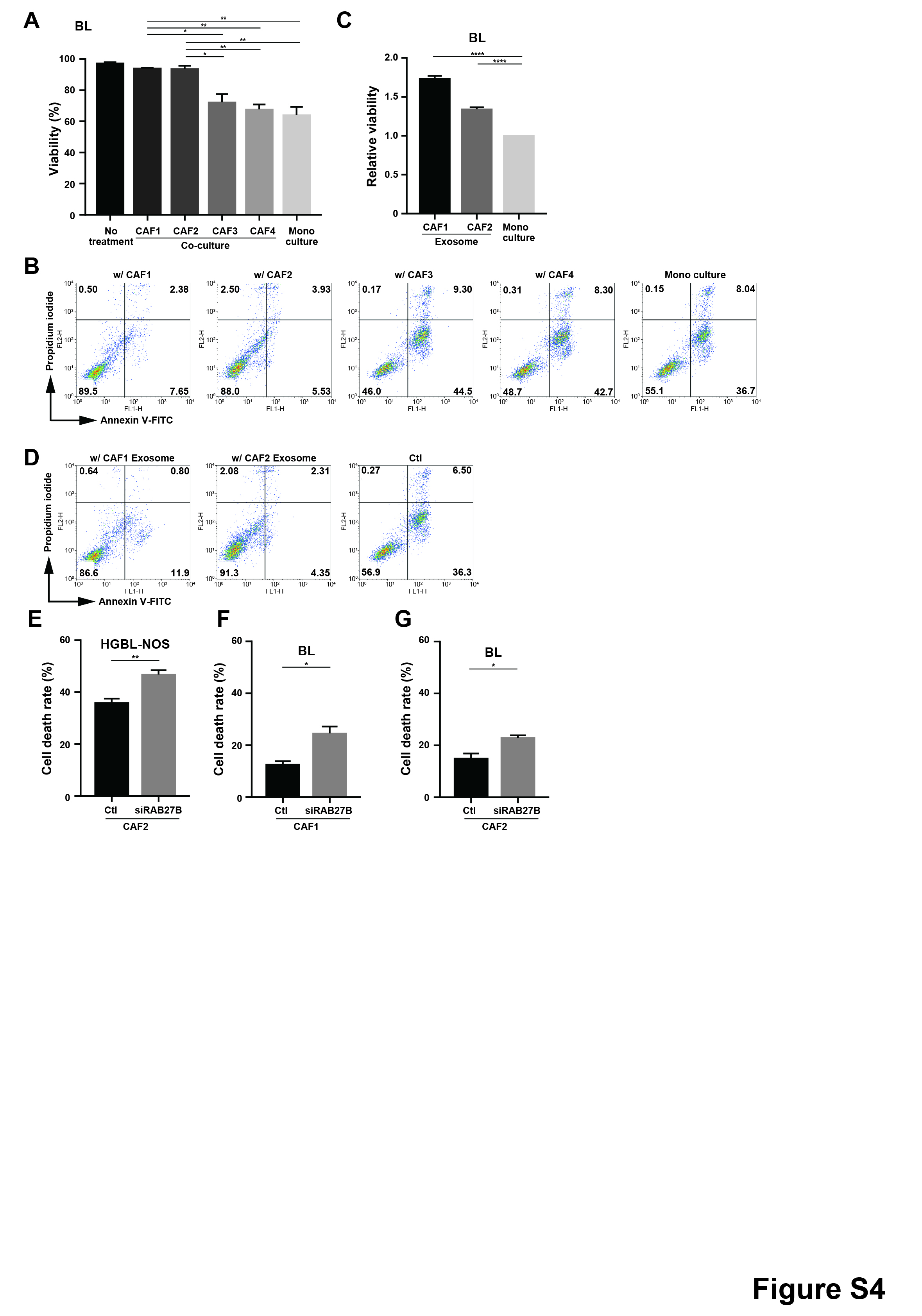

Supplement: Supplementary file 11 — Figure S4 [file 41388_2021_1829_MOESM11_ESM.jpg]

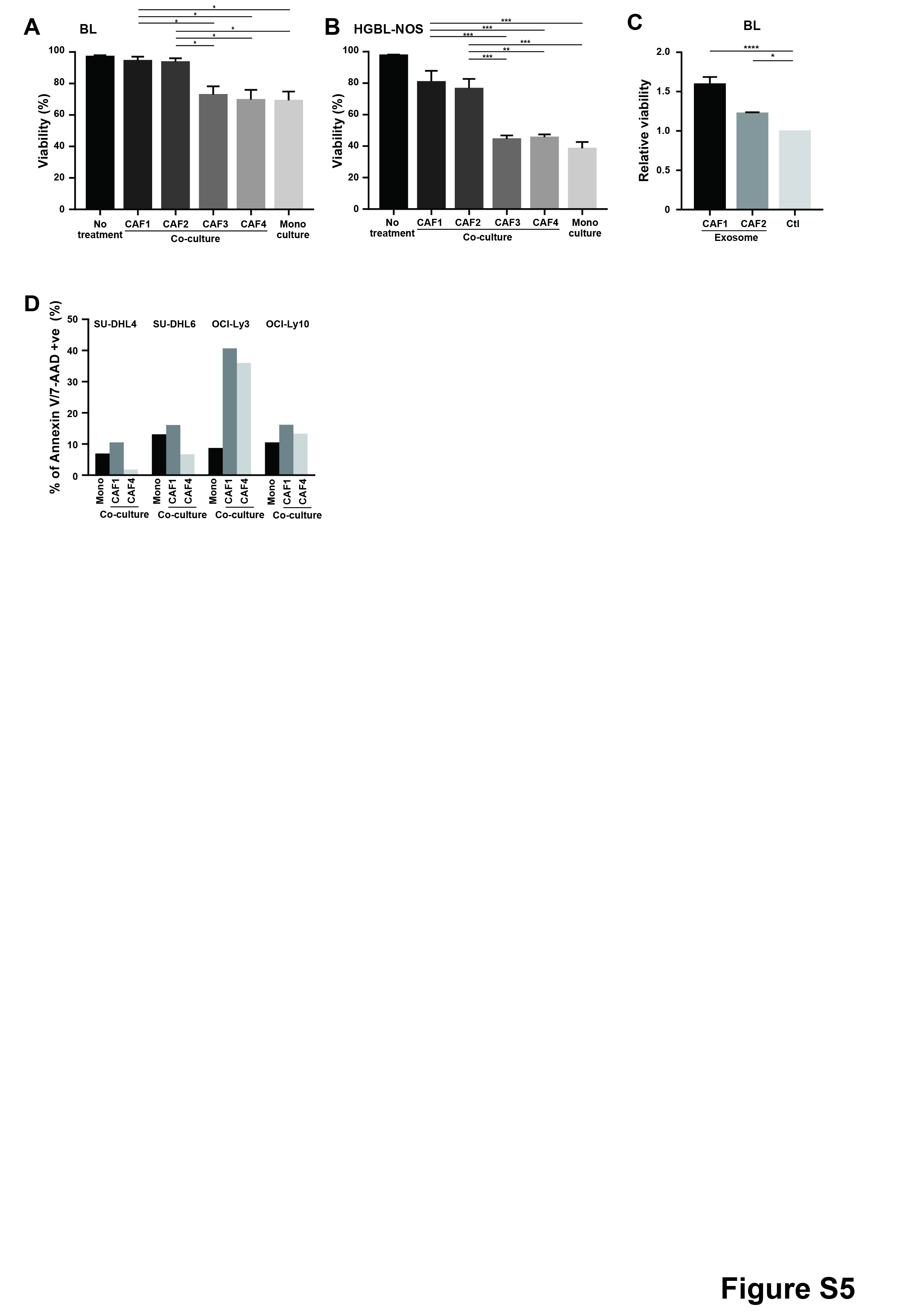

Supplement: Supplementary file 12 — Figure S5 [file 41388_2021_1829_MOESM12_ESM.jpg]

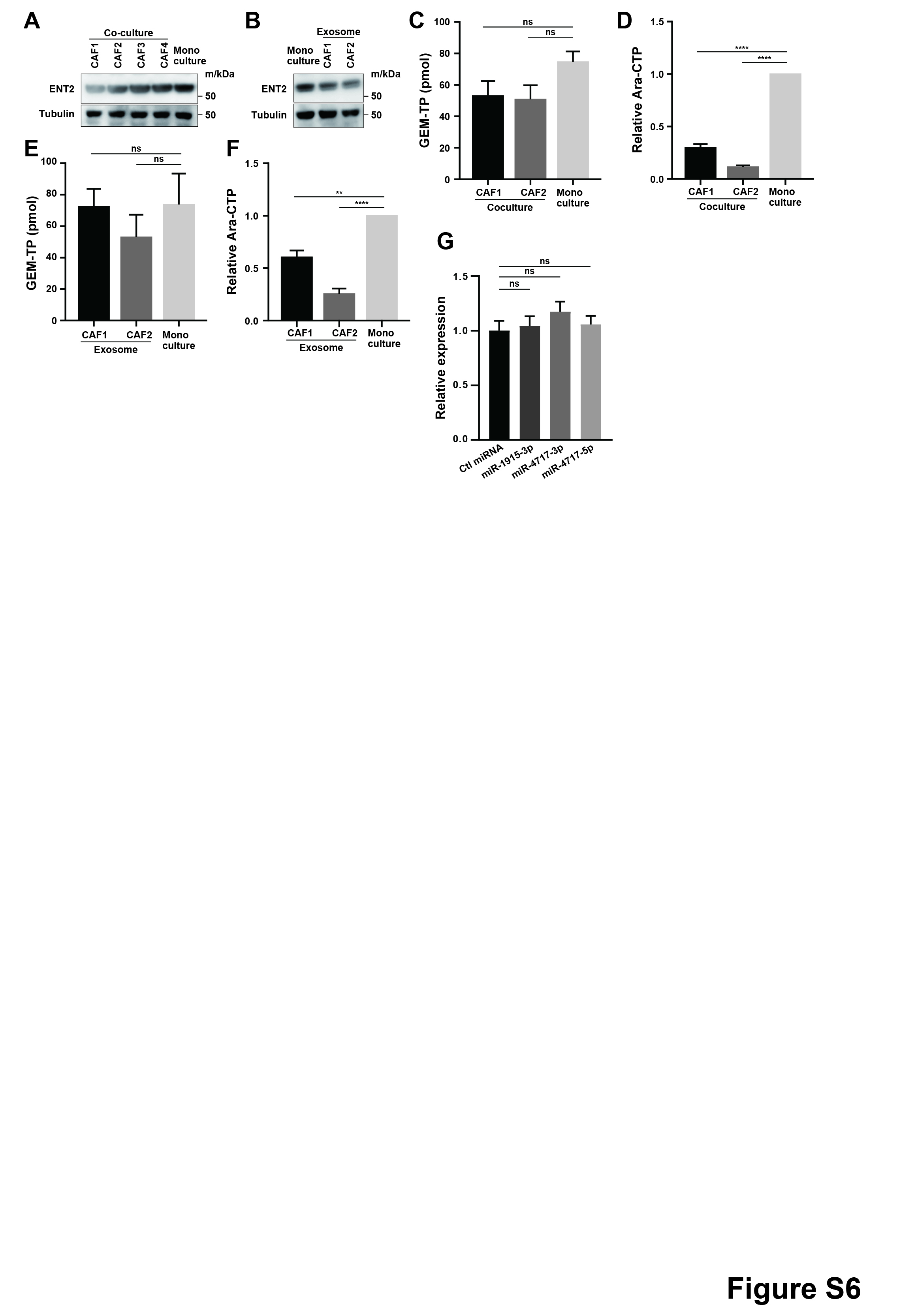

Supplement: Supplementary file 13 — Figure S6 [file 41388_2021_1829_MOESM13_ESM.jpg]

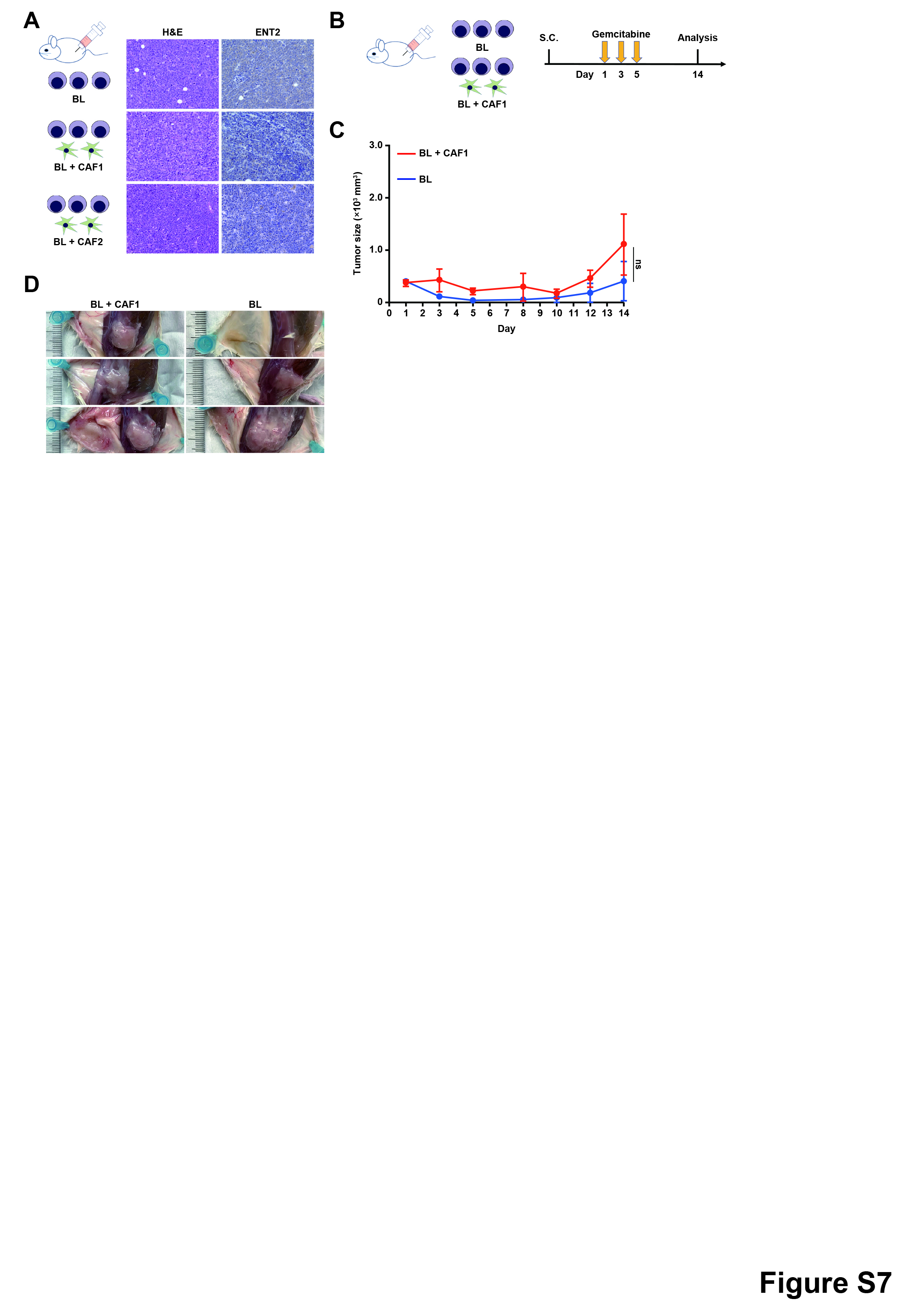

Supplement: Supplementary file 14 — Figure S7 [file 41388_2021_1829_MOESM14_ESM.jpg]

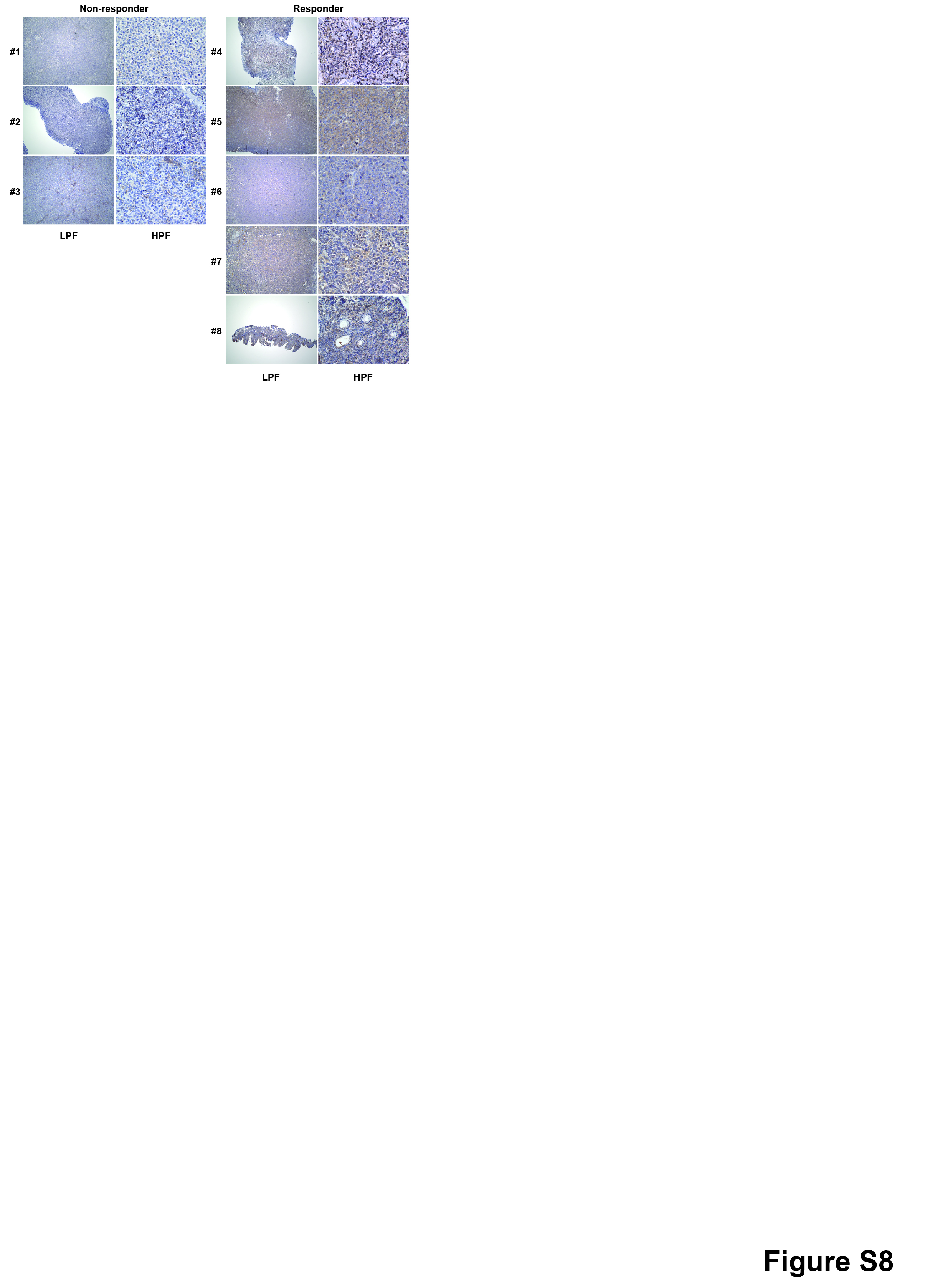

Supplement: Supplementary file 15 — Figure S8 [file 41388_2021_1829_MOESM15_ESM.jpg]
